# Physiological effects of foliar-applied β-carotene and carrot root extract on growth, biochemical traits, and yield of two flax varieties grown in sandy soil

**DOI:** 10.1186/s12870-026-09711-3

**Published:** 2026-08-18

**Authors:** AbdelSamad M. Younis, Mervet Sh. Sadak, Bakry A. Bakry, Omar M. Ibrahim, Amira M. El-Tahan

**Affiliations:** 1 Field Crops Research Department, Agricultural and Biological Research Institute, National Research Centre, P.O. 12622, 33 El Bohouth Street, Dokki, Giza, Egypt; 2 Botany Department, Agricultural and Biological Research Institute, National Research Centre, P.O. 12622, 33 El Bohouth Street, Dokki, Giza, Egypt; 3 Plant Production Department, Arid Lands Cultivation Research Institute (ALCRI), City of Scientific Research and Technological Applications (SRTA-City), New Borg El-Arab, Alexandria, Egypt

**Keywords:** *Linum usitatissimum* L., β-carotene, Carrot root extract, Foliar application, Sandy soil, Water deficit, Antioxidant-related traits, Seed yield

## Abstract

**Background:**

Flax (*Linum usitatissimum* L.) productivity in sandy soils is often constrained by limited water availability and reduced physiological performance. Although antioxidant-related foliar treatments may support crop performance, direct comparisons of purified β-carotene with carrot root extract, a natural multi-component biostimulant, remain scarce in flax. This field study evaluated the effects of foliar β-carotene and carrot root extract on growth, biochemical traits, and yield performance of two flax varieties, Sakha-2 and Line-4, grown in sandy soil under a field-imposed short-term water-deficit episode. The experiment was arranged in a split-plot design with three replicates. Varieties were assigned to main plots, while foliar treatments were assigned to subplots: untreated control, β-carotene at 25, 50, and 75 mg L⁻¹, and carrot root extract at 5, 10, and 20%.

**Results:**

Foliar treatments were associated with significant changes in growth, photosynthetic pigments, antioxidant-related traits, and yield attributes, and the magnitude of these responses differed between the two varieties. Across varieties, carrot root extract at 10% and β-carotene at 50 mg L⁻¹ produced the most consistent improvements across the measured traits. Compared with the untreated control, carrot extract at 10% increased shoot length from 45.16 to 64.50 cm, shoot fresh weight from 1.005 to 2.670 g, chlorophyll a from 1.020 to 1.635, total pigments from 1.875 to 2.755, and IAA from 17.51 to 42.34 µg g⁻¹ FW. It also enhanced phenolics, flavonoids, β-carotene content, DPPH radical-scavenging activity, PAL activity, and TAL activity. Yield responses followed the same trend: carrot extract at 10% increased seed yield from 945 to 1748 kg ha⁻¹, biological yield from 5112 to 9157 kg ha⁻¹, 1000-seed weight from 4.165 to 6.025 g, oil percentage from 32.01 to 33.43%, and oil yield from 301 to 584 kg ha⁻¹. β-carotene at 50 mg L⁻¹ also increased seed yield to 1723 kg ha⁻¹ and oil yield to 576 kg ha⁻¹.

**Conclusions:**

Foliar application of β-carotene and carrot root extract was associated with improvements in several physiological, biochemical, and yield-related traits of flax grown in sandy soil under a field-imposed short-term water-deficit episode. Carrot root extract at 10% produced the most consistent overall response across the measured traits, while β-carotene at 50 mg L⁻¹ was also associated with marked yield improvements. These findings suggest that both treatments may support flax performance under the conditions evaluated in the present study. However, further studies using quantitatively defined irrigation regimes, direct measurements of soil and plant water status, and comprehensive compound-level phytochemical profiling of carrot root extract are required to confirm the underlying mechanisms and optimum application concentrations.

**Supplementary Information:**

The online version contains supplementary material available at 10.1186/s12870-026-09711-3.

## Introduction

Climate change, increasing water scarcity, and the expansion of agriculture into marginal lands have intensified the exposure of field crops to abiotic stresses, particularly in arid and semi-arid regions. Among these constraints, limited water availability is a major factor restricting plant growth and crop productivity because it adversely affects cell expansion, photosynthetic activity, nutrient uptake, assimilate partitioning, and reproductive development [1–3]. These effects may be particularly pronounced in sandy soils because of their low water-holding capacity, limited nutrient retention, and rapid drying after irrigation. Consequently, crops cultivated in newly reclaimed sandy soils may require additional management strategies to maintain physiological performance and productivity during temporary or seasonal periods of limited water availability [1].

Flax (*Linum usitatissimum* L.) is an important multipurpose crop cultivated for seed, oil, fiber, and industrial uses. It is one of the oldest domesticated crops and remains valuable because of its dual economic importance as both an oilseed and fiber crop. Flaxseed is rich in oil, protein, dietary fiber, lignans, phenolic compounds, minerals, and essential fatty acids, particularly α-linolenic acid and linoleic acid, which support its nutritional and industrial value [4]. Flax oil is used not only as a high-quality edible oil but also in several industrial applications, including paints, varnishes, printing inks, and other bio-based products [4]. However, despite its adaptability, flax productivity is highly sensitive to unfavorable environmental conditions, especially water limitation during active vegetative growth and reproductive development.

Water deficit can impair flax productivity through several interconnected physiological and biochemical mechanisms. It reduces leaf expansion, biomass accumulation, photosynthetic pigment stability, and carbon assimilation, while promoting excessive formation of reactive oxygen species (ROS). Excessive ROS accumulation can damage membranes, proteins, pigments, and nucleic acids, ultimately reducing growth, seed filling, and oil production [1, 3]. In flax, previous studies showed that drought or water stress reduced growth, seed yield, and yield components, and that the magnitude of these reductions varied among genotypes and physiological traits [2, 3]. Such genotype-dependent responses indicate that improving flax performance under water-limited conditions requires strategies that strengthen physiological resilience rather than merely increasing vegetative growth.

Plants counteract water-deficit-induced oxidative stress through enzymatic and non-enzymatic antioxidant systems. Non-enzymatic antioxidants such as phenolic compounds, flavonoids, carotenoids, ascorbate, and glutathione contribute to ROS scavenging and protection of cellular structures. In addition, phenylpropanoid metabolism is central to stress adaptation because it supplies phenolics, flavonoids, lignin precursors, and other defense-related metabolites. Phenylalanine ammonia-lyase (PAL) and tyrosine ammonia-lyase (TAL) are key enzymes in this pathway and are commonly associated with enhanced antioxidant potential and improved stress tolerance [5]. Therefore, foliar treatments capable of maintaining photosynthetic pigments, improving antioxidant capacity, and stimulating phenylpropanoid-related metabolism may offer a practical approach for improving flax productivity in sandy soil under water-deficit conditions.

In recent years, foliar application of biostimulants and antioxidant-related compounds has gained attention as a sustainable strategy for improving crop performance under abiotic stress. Biostimulants can enhance plant resilience through several mechanisms, including modulation of antioxidant defense, osmotic adjustment, nutrient acquisition, hormonal balance, and stress-related gene expression [6–9]. In flax, foliar melatonin improved drought tolerance in two varieties grown in sandy soil by enhancing growth, antioxidant-related traits, phenolics, IAA, and yield [1]. Similarly, foliar thiourea improved flax performance under salt stress by reducing oxidative injury and strengthening antioxidant- and nutrient-related mechanisms [10]. More recently, foliar selenium improved antioxidant defense, yield, fatty acid composition, and mineral accumulation in flax [4], while foliar esculin and digitoxin enhanced growth and yield quality of salt-stressed flax by improving antioxidant defense and PAL/TAL-related responses [5]. These findings collectively demonstrate that flax can respond positively to foliar stress-mitigation treatments when such treatments support physiological and biochemical resilience.

Despite this progress, most available flax studies have focused on compounds such as melatonin, thiourea, selenium, esculin, digitoxin, microbial inoculants, or other stress-mitigation agents rather than comparing a purified carotenoid compound with a natural carotenoid-rich extract [1, 4, 5]. This distinction is important because a purified compound and a whole natural extract may not act through identical mechanisms. A purified compound may provide a targeted physiological effect, whereas a natural extract may contain multiple bioactive constituents that act additively or synergistically [11, 12]. Therefore, comparing β-carotene with carrot root extract may help clarify whether a single carotenoid-based foliar treatment or a multi-component natural extract is more effective for supporting flax performance under a field-imposed short-term water-deficit episode.

Carrot (*Daucus carota* L.) root is a rich source of bioactive compounds, including carotenoids, phenolic compounds, flavonoids, ascorbic acid, sugars, amino acids, and other redox-active metabolites. Carrot phytochemicals, particularly carotenoids and phenolics, have been widely recognized for their antioxidant properties and their contribution to plant and human health-related functions [13]. Recent studies also indicate that carrot-derived extracts may act as natural biostimulants under stress conditions. Teixeira et al. [14] reported that carrot extract seed priming improved seed performance and early physiological responses of rice under salinity and was associated with reduced oxidative stress. Earlier work by Kasim et al. [15] also suggested that carrot root extract could alleviate drought-induced growth inhibition in *Vicia faba*. These findings support the idea that carrot extract may provide bioactive molecules capable of modulating stress responses, although its foliar application in flax remains insufficiently explored.

β-Carotene is one of the most important carotenoids in plants and plays a central role in photosynthesis and photoprotection. It is associated with photosynthetic reaction centers and light-harvesting complexes and contributes to protection against photooxidative damage by quenching singlet oxygen and limiting free-radical-mediated injury [16, 17]. Under abiotic stress, carotenoid metabolism is closely linked with chlorophyll stability, ROS detoxification, and maintenance of photosynthetic efficiency. Babaei et al. [18] reported that foliar β-carotene mitigated salt stress in *Lepidium sativum* by reducing hydrogen peroxide and malondialdehyde, improving antioxidant enzyme activity, and increasing phenolic, glutathione, and chlorophyll contents. In addition, β-carotene-derived apocarotenoids such as β-cyclocitral have been reported to function as stress-related signaling molecules that regulate stress-responsive pathways in plants [19, 20]. These findings suggest that β-carotene may have both antioxidant and signaling-related roles under stress conditions.

Based on this background, the present study addresses a clear knowledge gap: direct evidence comparing purified β-carotene with carrot root extract as foliar treatments in flax grown in sandy soil under a field-imposed short-term water-deficit episode remains scarce. This comparison is relevant because β-carotene represents a targeted carotenoid-based antioxidant approach, whereas carrot root extract represents a natural multi-component biostimulant containing carotenoids together with phenolics, vitamins, and other metabolites. Moreover, the response to foliar treatments may differ between flax varieties because genotypes vary in pigment stability, antioxidant metabolism, phenolic accumulation, hormone balance, and yield-forming capacity under stress [1, 2]. Therefore, evaluating the responses of Sakha-2 and Line-4 to β-carotene and carrot root extract may provide useful insight into treatment-specific and variety-specific mechanisms of stress mitigation.

Accordingly, this study hypothesized that foliar application of β-carotene and carrot root extract would be associated with improved flax performance in sandy soil under a field-imposed short-term water-deficit episode, accompanied by changes in photosynthetic pigment accumulation, antioxidant-related metabolism, phenolic and flavonoid accumulation, IAA status, and yield formation. It was further hypothesized that the magnitude of response would differ between Sakha-2 and Line-4 because of varietal differences in physiological and biochemical capacity. Therefore, the objectives of this study were as follows (i) compare the growth and yield responses of two flax varieties to foliar β-carotene and carrot root extract, (ii) evaluate their effects on photosynthetic pigments, IAA, phenolic compounds, flavonoids, antioxidant activity, PAL, and TAL, and (iii) identify the most effective treatment–variety combination associated with improved flax productivity under sandy soil conditions.

## Results

### Varietal differences in growth, physiological traits, antioxidant-related metabolism, and yield

The two flax varieties differed significantly in most growth, physiological, biochemical, and yield-related traits (Table 1). Sakha-2 showed higher vegetative growth than Line-4, with shoot length increasing from 51.67 cm in Line-4 to 64.67 cm in Sakha-2, representing a 25.2% increase. Shoot fresh weight, root length, and root fresh weight were also higher in Sakha-2 by 64.1, 20.6, and 93.0%, respectively, compared with Line-4. These results indicate that Sakha-2 exhibited greater vegetative and root growth than Line-4 in sandy soil under the field-imposed short-term water-deficit episode.

**Table 1:** Varietal differences in growth, physiological, biochemical, and yield-related traits of flax

Trait	Sakha-2	Line-4
Shoot length (cm)	64.67 ± 2.54 a	51.67 ± 1.23 b
Shoot fresh weight (g)	2.553 ± 0.261 a	1.556 ± 0.123 b
Root length (cm)	11.14 ± 0.26 a	9.24 ± 0.21 b
Root fresh weight (g)	0.276 ± 0.028 a	0.143 ± 0.012 b
Chlorophyll a (mg g⁻¹ FW)	1.417 ± 0.044 a	1.263 ± 0.040 b
Chlorophyll b (mg g⁻¹ FW)	0.689 ± 0.011 a	0.649 ± 0.011 b
Carotenoids (mg g⁻¹ FW)	0.344 ± 0.008 b	0.361 ± 0.011 a
Total pigments (mg g⁻¹ FW)	2.450 ± 0.062 a	2.271 ± 0.056 b
IAA (µg g⁻¹ FW)	24.83 ± 1.90 b	33.94 ± 1.85 a
Phenolics (mg 100 g⁻¹ DW)	57.58 ± 1.88 a	53.24 ± 1.85 b
Flavonoids (mg 100 g⁻¹ DW)	22.24 ± 0.56 a	22.02 ± 1.07 b
β-carotene (mg 100 g⁻¹ DW)	0.186 ± 0.008 a	0.180 ± 0.004 a
DPPH (%)	35.07 ± 0.39 b	45.89 ± 0.62 a
PAL	2.443 ± 0.101 a	2.115 ± 0.081 b
TAL	6.480 ± 0.109 a	5.320 ± 0.113 b
Technical stem length (cm)	62.14 ± 0.86 b	66.24 ± 1.91 a
Fruiting zone length (cm)	21.67 ± 0.42 a	21.19 ± 1.58 b
Plant height (cm)	83.81 ± 1.13 b	87.43 ± 2.74 a
No. of fruiting branches plant⁻¹	12.05 ± 0.42 a	10.57 ± 0.86 b
No. of capsules plant⁻¹	41.95 ± 2.27 b	51.29 ± 2.00 a
Seed yield (g plant⁻¹)	3.369 ± 0.077 b	3.927 ± 0.243 a
Biological yield (g plant⁻¹)	6.984 ± 0.310 a	6.103 ± 0.252 b
Straw yield (kg ha⁻¹)	6534 ± 395 a	6145 ± 391 b
Seed yield (kg ha⁻¹)	1315 ± 59 b	1541 ± 61 a
Biological yield (kg ha⁻¹)	7854 ± 440 a	7681 ± 426 b
1000-seed weight (g)	5.181 ± 0.116 b	5.633 ± 0.185 a
Oil (%)	34.18 ± 0.13 a	31.84 ± 0.23 b
Oil yield (kg ha⁻¹)	450 ± 21 b	492 ± 20 a

Values are means ± SE. Different letters within the same column indicate significant differences according to Tukey’s HSD test at *p* ≤ 0.05

Photosynthetic pigment traits also differed between the two varieties. Sakha-2 recorded higher chlorophyll a, chlorophyll b, and total pigments than Line-4. Chlorophyll a increased from 1.263 in Line-4 to 1.417 in Sakha-2, while total pigments increased from 2.271 to 2.450. In contrast, carotenoid content was slightly higher in Line-4 than in Sakha-2, with values of 0.361 and 0.344 mg g⁻¹ FW, respectively. Endogenous IAA showed the same Line-4-dominant pattern, being higher in Line-4 than in Sakha-2, with values of 33.94 and 24.83 µg g⁻¹ FW, respectively.

The two varieties also differed in antioxidant-related traits. Sakha-2 had higher phenolic content, PAL activity, and TAL activity than Line-4, whereas Line-4 showed markedly higher DPPH radical-scavenging activity. Phenolic content increased from 53.24 mg 100 g⁻¹ DW in Line-4 to 57.58 mg 100 g⁻¹ DW in Sakha-2. Similarly, PAL and TAL activities were higher in Sakha-2 by 15.6 and 21.8%, respectively. Conversely, DPPH activity was 45.89% in Line-4 compared with 35.07% in Sakha-2. Flavonoids were slightly higher in Sakha-2 than in Line-4, whereas β-carotene content showed only a marginal varietal difference. 

Yield and yield components showed variety-dependent responses. Sakha-2 had higher values for the number of fruiting branches per plant, biological yield per plant, straw yield, biological yield per hectare, and oil percentage. In contrast, Line-4 recorded higher technical stem length, plant height, number of capsules per plant, seed yield per plant, seed yield per hectare, 1000-seed weight, and oil yield. Seed yield increased from 1315 kg ha⁻¹ in Sakha-2 to 1541 kg ha⁻¹ in Line-4, while oil yield increased from 450 to 492 kg ha⁻¹. These results suggest that Sakha-2 had stronger vegetative and biomass-forming capacity, whereas Line-4 showed better reproductive efficiency and seed yield formation.

### Effects of foliar β-carotene and carrot root extract on growth traits

Foliar application of β-carotene and carrot root extract significantly affected all measured growth traits (Table 2). Compared with the untreated control, all foliar treatments improved shoot length, shoot fresh weight, root length, and root fresh weight, but the magnitude of response differed among concentrations. Carrot extract at 20% produced the highest shoot length and shoot fresh weight, increasing shoot length from 45.16 to 66.17 cm, equivalent to a 46.5% increase, and shoot fresh weight from 1.005 to 3.210 g, equivalent to a 219.4% increase. Carrot extract at 10% also showed a strong response, increasing shoot length by 42.8% and shoot fresh weight by 165.7% relative to the control.

**Table 2:** Effects of foliar β-carotene and carrot root extract on flax growth traits

Treatment	Shoot length (cm)	Shoot fresh weight (g)	Root length (cm)	Root fresh weight (g)
Control	45.16 ± 0.98 b	1.005 ± 0.117 c	8.67 ± 0.45 d	0.095 ± 0.012 e
β-carotene 25 mg L⁻¹	53.66 ± 1.99 b	1.205 ± 0.052 c	9.16 ± 0.38 d	0.125 ± 0.016 e
β-carotene 50 mg L⁻¹	60.66 ± 2.63 a	2.125 ± 0.194 b	9.83 ± 0.23 c	0.190 ± 0.007 d
β-carotene 75 mg L⁻¹	57.50 ± 5.14 a	2.300 ± 0.158 b	11.84 ± 0.55 a	0.205 ± 0.054 c
Carrot extract 5%	59.50 ± 4.39 a	1.865 ± 0.320 b	10.50 ± 0.55 b	0.235 ± 0.016 c
Carrot extract 10%	64.50 ± 4.24 a	2.670 ± 0.348 a	11.00 ± 0.45 b	0.330 ± 0.058 a
Carrot extract 20%	66.17 ± 5.90 a	3.210 ± 0.696 a	10.33 ± 0.46 c	0.285 ± 0.060 b

Values are means ± SE. Different letters within the same column indicate significant differences according to Tukey’s HSD test at *p* ≤ 0.05

Root growth responded differently from shoot growth. β-carotene at 75 mg L⁻¹ produced the highest root length, increasing it from 8.67 to 11.84 cm, equivalent to a 36.6% increase compared with the untreated control. In contrast, carrot extract at 10% produced the highest root fresh weight, increasing it from 0.095 to 0.330 g. These results indicate that carrot extract was more effective in promoting shoot biomass and root fresh weight, whereas the highest β-carotene concentration was more effective in enhancing root elongation.

### Effects of foliar treatments on photosynthetic pigments and IAA

Foliar treatments significantly improved chlorophyll a, chlorophyll b, carotenoids, total pigments, and IAA content (Table 3). Carrot extract at 10% produced the highest values for most pigment traits and IAA. Compared with the untreated control, carrot extract at 10% increased chlorophyll a from 1.02 to 1.64, chlorophyll b from 0.58 to 0.73, carotenoids from 0.28 to 0.39, and total pigments from 1.87 to 2.76. These increases corresponded to 60.3, 25.9, 43.6, and 46.9%, respectively.

**Table 3:** Effects of foliar β-carotene and carrot root extract on photosynthetic pigments and IAA in flax

Treatment	Chlorophyll a (mg g⁻¹ FW)	Chlorophyll b (mg g⁻¹ FW)	Carotenoids (mg g⁻¹ FW)	Total pigments (mg g⁻¹ FW)	IAA (µg g⁻¹ FW)
Control	1.020 ± 0.026 e	0.580 ± 0.016 d	0.275 ± 0.007 e	1.875 ± 0.047 e	17.51 ± 2.02 d
β-carotene 25 mg L⁻¹	1.200 ± 0.036 d	0.650 ± 0.009 c	0.340 ± 0.009 d	2.190 ± 0.032 d	25.46 ± 2.96 c
β-carotene 50 mg L⁻¹	1.430 ± 0.063 b	0.685 ± 0.002 b	0.385 ± 0.007 a	2.500 ± 0.061 b	36.70 ± 2.21 b
β-carotene 75 mg L⁻¹	1.330 ± 0.032 c	0.690 ± 0.010 b	0.375 ± 0.020 b	2.390 ± 0.010 c	29.68 ± 2.44 c
Carrot extract 5%	1.300 ± 0.018 c	0.670 ± 0.009 b	0.345 ± 0.007 c	2.320 ± 0.024 c	21.96 ± 0.95 d
Carrot extract 10%	1.635 ± 0.029 a	0.730 ± 0.023 a	0.395 ± 0.002 a	2.755 ± 0.053 a	42.34 ± 2.30 a
Carrot extract 20%	1.465 ± 0.052 b	0.675 ± 0.020 b	0.355 ± 0.008 c	2.495 ± 0.078 b	32.02 ± 3.09 b

Values are means ± SE. Different letters within the same column indicate significant differences according to Tukey’s HSD test at *p* ≤ 0.05

IAA content showed an even stronger response to foliar treatments. Carrot extract at 10% increased IAA from 17.51 to 42.34 µg g⁻¹ FW, representing a 141.8% increase compared with the control. β-carotene at 50 mg L⁻¹ ranked second, increasing IAA to 36.70 µg g⁻¹ FW, equivalent to a 109.6% increase. These results suggest that the improved growth response under carrot extract at 10% may be associated with enhanced pigment accumulation and higher endogenous auxin status.

### Effects of foliar treatments on antioxidant compounds, DPPH activity, PAL, and TAL

Foliar application of β-carotene and carrot extract significantly affected all antioxidant-related traits of flax plants, including phenolic content, flavonoid content, β-carotene content, DPPH radical-scavenging activity, PAL activity, and TAL activity (Table 4). Among the applied treatments, carrot extract at 10% produced the strongest overall antioxidant response. It recorded the highest phenolic content, flavonoid content, DPPH activity, PAL activity, and TAL activity, with values of 66.94 mg 100 g⁻¹ DW, 27.28 mg 100 g⁻¹ DW, 42.77%, 2.845, and 6.448, respectively. Compared with the untreated control, carrot extract at 10% increased phenolic content from 41.42 to 66.94 mg 100 g⁻¹ DW and flavonoid content from 17.15 to 27.28 mg 100 g⁻¹ DW. The same treatment also increased DPPH activity from 36.96 to 42.77%, PAL activity from 1.495 to 2.845, and TAL activity from 5.020 to 6.448. For β-carotene content, carrot extract at 20% recorded the highest value of 0.217, followed closely by carrot extract at 10% with 0.215, compared with 0.150 in the untreated control. β-carotene at 50 mg L⁻¹ also enhanced antioxidant-related traits, recording 58.56 mg 100 g⁻¹ DW phenolic content, 23.99 mg 100 g⁻¹ DW flavonoid content, 42.12% DPPH activity, 2.515 PAL activity, and 6.108 TAL activity.

**Table 4:** Effects of foliar β-carotene and carrot root extract on antioxidant-related traits of flax

Treatment	Phenolics (mg 100 g⁻¹ DW)	Flavonoids (mg 100 g⁻¹ DW)	β-carotene (mg 100 g⁻¹ DW)	DPPH (%)	PAL	TAL
Control	41.41 ± 1.04 d	17.15 ± 0.69 c	0.150 ± 0.000 c	36.96 ± 1.95 d	1.495 ± 0.065 f	5.020 ± 0.291 e
β-carotene 25 mg L⁻¹	50.24 ± 2.01 c	20.39 ± 0.88 b	0.165 ± 0.004 b	38.56 ± 2.31 c	2.052 ± 0.036 e	5.537 ± 0.252 d
β-carotene 50 mg L⁻¹	58.56 ± 2.26 b	23.99 ± 0.25 a	0.185 ± 0.007 b	42.12 ± 3.03 a	2.515 ± 0.070 b	6.108 ± 0.333 b
β-carotene 75 mg L⁻¹	59.06 ± 1.62 b	19.86 ± 1.10 b	0.173 ± 0.006 b	40.81 ± 2.58 b	2.152 ± 0.024 d	6.188 ± 0.140 b
Carrot extract 5%	53.27 ± 1.71 b	21.66 ± 1.03 b	0.175 ± 0.004 b	40.33 ± 2.48 b	2.400 ± 0.121 c	5.785 ± 0.270 c
Carrot extract 10%	66.94 ± 0.86 a	27.28 ± 1.35 a	0.215 ± 0.006 a	42.77 ± 2.66 a	2.845 ± 0.123 a	6.448 ± 0.337 a
Carrot extract 20%	58.36 ± 2.99 b	24.59 ± 1.02 a	0.217 ± 0.015 a	41.81 ± 2.24 a	2.493 ± 0.093 b	6.212 ± 0.244 b

Values are means ± SE. Different letters within the same column indicate significant differences according to Tukey’s HSD test at *p* ≤ 0.05

### Effects of foliar treatments on yield components, seed yield, and oil yield

Foliar treatments significantly affected yield components, seed yield, biological yield, oil percentage, and oil yield (Tables 5 and 6). Carrot extract at 10% produced the highest technical stem length, plant height, number of capsules per plant, seed yield per plant, biological yield per plant, 1000-seed weight, seed yield per hectare, and oil yield. Compared with the untreated control, carrot extract at 10% increased technical stem length from 53.84 to 72.50 cm, plant height from 73.67 to 95.50 cm, number of capsules per plant from 32.34 to 62.34, seed yield per plant from 2.875 to 4.675 g, and biological yield per plant from 4.655 to 8.470 g (Table 5). At the field scale, the same treatment increased seed yield from 945 to 1748 kg ha⁻¹, biological yield from 5112 to 9157 kg ha⁻¹, oil percentage from 32.01 to 33.43%, and oil yield from 301 to 584 kg ha⁻¹ (Table 6). β-carotene at 50 mg L⁻¹ also showed a strong yield response, recording 1723 kg ha⁻¹ seed yield, 10,080 kg ha⁻¹ biological yield, and 576 kg ha⁻¹ oil yield. Carrot extract at 20% produced the highest number of fruiting branches per plant and the highest fruiting zone length, but it did not produce the highest seed yield, biological yield per plant, or oil yield (Tables 5 and 6).

**Table 5:** Effects of foliar β-carotene and carrot root extract on yield components of flax

Treatment	Technical stem length (cm)	Fruiting zone length (cm)	Plant height (cm)	No. of fruiting branches plant⁻¹	No. of capsules plant⁻¹	Seed yield (g plant⁻¹)	Biological yield (g plant⁻¹)
Control	53.84 ± 1.44 d	19.84 ± 0.83 c	73.67 ± 1.22 c	9.67 ± 0.09 c	32.34 ± 3.92 d	2.875 ± 0.075 c	4.655 ± 0.023 d
β-carotene 25 mg L⁻¹	62.16 ± 0.72 c	15.00 ± 2.70 d	77.17 ± 2.21 c	9.33 ± 0.49 c	42.17 ± 3.08 c	3.090 ± 0.155 b	6.435 ± 0.235 b
β-carotene 50 mg L⁻¹	63.34 ± 0.48 c	21.67 ± 0.80 b	85.00 ± 1.51 b	10.33 ± 0.91 b	53.33 ± 2.05 b	3.475 ± 0.085 b	7.790 ± 0.301 a
β-carotene 75 mg L⁻¹	61.00 ± 1.25 c	25.67 ± 1.37 a	86.67 ± 2.40 b	13.34 ± 0.75 a	44.00 ± 2.06 c	3.465 ± 0.041 b	6.465 ± 0.334 b
Carrot extract 5%	68.17 ± 2.28 b	19.00 ± 1.34 c	87.17 ± 1.22 b	9.50 ± 1.58 c	43.16 ± 0.98 c	3.710 ± 0.256 b	5.465 ± 0.226 c
Carrot extract 10%	72.50 ± 2.54 a	23.00 ± 0.65 b	95.50 ± 3.14 a	11.84 ± 1.28 b	62.34 ± 2.65 a	4.675 ± 0.440 a	8.470 ± 0.384 a
Carrot extract 20%	68.33 ± 2.59 a	25.83 ± 2.61 a	94.17 ± 4.89 a	15.17 ± 1.38 a	49.00 ± 3.04 b	4.245 ± 0.492 a	6.525 ± 0.216 b

Values are means ± SE. Different letters within the same column indicate significant differences according to Tukey’s HSD test at *p* ≤ 0.05

**Table 6:** Effects of foliar β-carotene and carrot root extract on seed yield, oil yield, and seed composition of flax

Treatment	Straw yield (kg ha⁻¹)	Seed yield (kg ha⁻¹)	Biological yield (kg ha⁻¹)	1000-seed weight (g)	Oil (%)	Oil yield (kg ha⁻¹)
Control	4165 ± 337 c	945 ± 38 d	5112 ± 389 d	4.165 ± 0.158 c	32.01 ± 0.70 c	301 ± 6 d
β-carotene 25 mg L⁻¹	7715 ± 190 a	1360 ± 103 b	9072 ± 282 b	5.190 ± 0.060 b	32.91 ± 0.52 b	445 ± 27 b
β-carotene 50 mg L⁻¹	8353 ± 217 a	1723 ± 56 a	10,080 ± 319 a	5.820 ± 0.153 a	33.50 ± 0.56 a	576 ± 17 a
β-carotene 75 mg L⁻¹	4933 ± 405 c	1297 ± 26 c	6228 ± 385 c	5.740 ± 0.320 a	33.20 ± 0.43 b	430 ± 3 c
Carrot extract 5%	4463 ± 169 c	1485 ± 68 b	5952 ± 143 c	5.280 ± 0.145 b	32.01 ± 0.72 c	473 ± 15 b
Carrot extract 10%	7415 ± 525 b	1748 ± 56 a	9157 ± 448 b	6.025 ± 0.129 a	33.43 ± 0.44 a	584 ± 9 a
Carrot extract 20%	7332 ± 222 b	1438 ± 68 b	8775 ± 127 b	5.630 ± 0.292 a	34.02 ± 0.44 a	488 ± 21 b

Values are means ± SE. Different letters within the same column indicate significant differences according to Tukey’s HSD test at *p* ≤ 0.05

### Treatment × variety interaction effects on growth traits

The interaction between variety and foliar treatment significantly affected all growth traits (Table 7). The strongest shoot growth response was observed in Sakha-2 treated with carrot extract at 20%, which recorded the highest shoot length and shoot fresh weight, with values of 78.67 cm and 4.66 g, respectively. These values were substantially higher than the untreated control of the same variety, which recorded 44.33 cm and 1.21 g. Sakha-2 treated with carrot extract at 10% ranked second for shoot length and produced the highest root fresh weight, reaching 73.33 cm and 0.46 g, respectively.

**Table 7:** Interaction effect between flax variety and foliar treatment on growth traits

Treatment	Shoot length (cm)	Shoot fresh weight (g)	Root length (cm)	Root fresh weight (g)
Control × Sakha-2	44.33 ± 1.14 b	1.210 ± 0.150 c	9.67 ± 0.06 d	0.120 ± 0.000 d
Control × Line-4	46.00 ± 1.68 b	0.800 ± 0.058 d	7.67 ± 0.03 e	0.070 ± 0.012 d
β-carotene 25 mg L⁻¹ × Sakha-2	56.00 ± 0.30 b	1.320 ± 0.012 c	10.00 ± 0.10 c	0.160 ± 0.000 c
β-carotene 25 mg L⁻¹ × Line-4	51.33 ± 3.77 b	1.090 ± 0.012 c	8.33 ± 0.02 e	0.090 ± 0.006 d
β-carotene 50 mg L⁻¹ × Sakha-2	65.00 ± 0.61 a	2.070 ± 0.150 c	10.33 ± 0.10 c	0.180 ± 0.012 c
β-carotene 50 mg L⁻¹ × Line-4	56.33 ± 3.94 b	2.180 ± 0.404 c	9.33 ± 0.07 d	0.200 ± 0.006 c
β-carotene 75 mg L⁻¹ × Sakha-2	66.67 ± 4.71 a	2.640 ± 0.006 b	13.00 ± 0.38 a	0.320 ± 0.035 b
β-carotene 75 mg L⁻¹ × Line-4	48.33 ± 5.11 b	1.960 ± 0.098 c	10.67 ± 0.09 c	0.090 ± 0.000 d
Carrot extract 5% × Sakha-2	68.67 ± 3.46 a	2.580 ± 0.029 b	11.67 ± 0.31 b	0.270 ± 0.006 b
Carrot extract 5% × Line-4	50.33 ± 0.42 b	1.150 ± 0.006 c	9.33 ± 0.18 d	0.200 ± 0.000 c
Carrot extract 10% × Sakha-2	73.33 ± 2.90 a	3.390 ± 0.294 b	12.00 ± 0.08 b	0.460 ± 0.006 a
Carrot extract 10% × Line-4	55.67 ± 1.83 b	1.950 ± 0.040 c	10.00 ± 0.08 c	0.200 ± 0.006 c
Carrot extract 20% × Sakha-2	78.67 ± 4.20 a	4.660 ± 0.543 a	11.33 ± 0.02 b	0.420 ± 0.006 a
Carrot extract 20% × Line-4	53.67 ± 0.18 b	1.760 ± 0.150 c	9.33 ± 0.23 d	0.150 ± 0.000 c

Values are means ± SE. Different letters within the same column indicate significant differences according to Tukey’s HSD test at *p* ≤ 0.05

Root length showed a different interaction pattern. The highest root length was recorded in Sakha-2 treated with β-carotene at 75 mg L⁻¹, reaching 13.00 cm, compared with 9.67 cm in untreated Sakha-2. In Line-4, the strongest growth responses were generally lower than those observed in Sakha-2, although β-carotene at 50 mg L⁻¹ and carrot extract at 10% improved shoot fresh weight and root fresh weight compared with the untreated Line-4 control. These results confirm that Sakha-2 was more responsive than Line-4 in vegetative growth traits, particularly under carrot extract treatments.

### Treatment × variety interaction effects on photosynthetic pigments, IAA, and oil percentage

The interaction between variety and foliar treatment significantly affected photosynthetic pigments, IAA, and oil percentage (Table 8). The highest chlorophyll a, chlorophyll b, and total pigment contents were recorded in Sakha-2 treated with carrot extract at 10%, with values of 1.70, 0.78, and 2.87, respectively. These values were higher than the corresponding untreated Sakha-2 control, which recorded 1.07, 0.61, and 1.97. Carrot extract at 10% also produced high pigment values in Line-4, but these remained slightly lower than those observed in Sakha-2.

**Table 8:** Interaction effect between flax variety and foliar treatment on photosynthetic pigments, IAA and oil

Treatment	Chlorophyll a (mg g⁻¹ FW)	Chlorophyll b (mg g⁻¹ FW)	Carotenoids (mg g⁻¹ FW)	Total pigments (mg g⁻¹ FW)	IAA (µg g⁻¹ FW)	Oil (%)
Control × Sakha-2	1.070 ± 0.000 e	0.610 ± 0.012 d	0.290 ± 0.006 f	1.970 ± 0.029 e	13.31 ± 1.66 d	33.57 ± 0.01 b
Control × Line-4	0.970 ± 0.029 f	0.550 ± 0.017 e	0.260 ± 0.000 g	1.780 ± 0.035 f	21.71 ± 0.20 c	30.44 ± 0.18 d
β-carotene 25 mg L⁻¹ × Sakha-2	1.280 ± 0.012 d	0.660 ± 0.000 c	0.320 ± 0.000 e	2.260 ± 0.006 c	18.87 ± 0.36 c	34.07 ± 0.04 a
β-carotene 25 mg L⁻¹ × Line-4	1.120 ± 0.006 e	0.640 ± 0.017 d	0.360 ± 0.000 c	2.120 ± 0.012 d	32.06 ± 0.36 b	31.75 ± 0.11 c
β-carotene 50 mg L⁻¹ × Sakha-2	1.560 ± 0.052 b	0.690 ± 0.000 b	0.370 ± 0.000 c	2.620 ± 0.064 b	32.14 ± 1.72 b	34.66 ± 0.11 a
β-carotene 50 mg L⁻¹ × Line-4	1.300 ± 0.006 c	0.680 ± 0.000 c	0.400 ± 0.000 b	2.380 ± 0.017 c	41.26 ± 0.78 a	32.34 ± 0.43 c
β-carotene 75 mg L⁻¹ × Sakha-2	1.400 ± 0.017 c	0.670 ± 0.000 c	0.330 ± 0.000 d	2.400 ± 0.017 c	24.28 ± 0.69 c	34.10 ± 0.33 a
β-carotene 75 mg L⁻¹ × Line-4	1.260 ± 0.006 d	0.710 ± 0.012 b	0.420 ± 0.006 a	2.380 ± 0.012 c	35.09 ± 0.03 b	32.30 ± 0.06 c
Carrot extract 5% × Sakha-2	1.340 ± 0.006 c	0.690 ± 0.000 b	0.330 ± 0.000 d	2.370 ± 0.012 c	20.07 ± 0.19 c	33.57 ± 0.21 b
Carrot extract 5% × Line-4	1.260 ± 0.006 d	0.650 ± 0.000 c	0.360 ± 0.000 c	2.270 ± 0.012 c	23.85 ± 0.95 c	30.44 ± 0.36 d
Carrot extract 10% × Sakha-2	1.700 ± 0.000 a	0.780 ± 0.000 a	0.400 ± 0.000 b	2.870 ± 0.006 a	39.86 ± 2.93 a	34.34 ± 0.32 a
Carrot extract 10% × Line-4	1.570 ± 0.006 b	0.680 ± 0.006 c	0.390 ± 0.000 b	2.640 ± 0.023 b	44.82 ± 3.42 a	32.52 ± 0.23 b
Carrot extract 20% × Sakha-2	1.570 ± 0.000 b	0.720 ± 0.000 b	0.370 ± 0.000 c	2.660 ± 0.029 b	25.29 ± 1.15 c	34.98 ± 0.17 a
Carrot extract 20% × Line-4	1.360 ± 0.052 c	0.630 ± 0.000 d	0.340 ± 0.012 d	2.330 ± 0.046 c	38.76 ± 0.98 a	33.06 ± 0.10 b

Values are means ± SE. Different letters within the same column indicate significant differences according to Tukey’s HSD test at *p* ≤ 0.05

Carotenoids and IAA showed different patterns. The highest carotenoid content was observed in Line-4 treated with β-carotene at 75 mg L⁻¹, reaching 0.42. In contrast, the highest IAA content was recorded in Line-4 treated with carrot extract at 10%, reaching 44.82 µg g⁻¹ FW, followed by Line-4 treated with β-carotene at 50 mg L⁻¹, which recorded 41.26 µg g⁻¹ FW. Oil percentage also showed a significant interaction response, with the highest values recorded in Sakha-2 treated with carrot extract at 20% and Sakha-2 treated with β-carotene at 50 mg L⁻¹. These results indicate that Sakha-2 was more responsive in pigment accumulation, whereas Line-4 was more responsive in IAA accumulation under the most effective foliar treatments.

### Treatment × variety interaction effects on antioxidant-related traits

The interaction between variety and foliar treatment significantly affected all antioxidant-related traits (Table 9). Phenolic content and PAL and TAL activities were highest in Sakha-2 treated with carrot extract at 10%, with values of 68.32 mg 100 g⁻¹ DW, 3.110, and 7.190, respectively. Compared with the untreated Sakha-2 control, these values represented substantial increases in phenylpropanoid-related metabolism and antioxidant-associated activity.

**Table 9:** Interaction effect between flax variety and foliar treatment on antioxidant-related traits

Treatment	Phenolics (mg 100 g⁻¹ DW)	Flavonoids (mg 100 g⁻¹ DW)	β-carotene (mg 100 g⁻¹ DW)	DPPH (%)	PAL	TAL
Control × Sakha-2	42.93 ± 1.31 c	18.61 ± 0.45 b	0.150 ± 0.000 c	32.61 ± 0.27 e	1.640 ± 0.006 f	5.670 ± 0.035 d
Control × Line-4	39.90 ± 1.18 c	15.69 ± 0.25 c	0.150 ± 0.000 c	41.31 ± 0.24 c	1.350 ± 0.006 g	4.370 ± 0.023 f
β-carotene 25 mg L⁻¹ × Sakha-2	54.19 ± 2.08 b	21.08 ± 1.42 b	0.163 ± 0.009 c	33.45 ± 0.71 e	2.130 ± 0.017 d	6.100 ± 0.000 c
β-carotene 25 mg L⁻¹ × Line-4	46.29 ± 0.56 b	19.70 ± 1.18 b	0.167 ± 0.003 c	43.67 ± 0.21 b	1.973 ± 0.015 e	4.973 ± 0.026 e
β-carotene 50 mg L⁻¹ × Sakha-2	63.33 ± 1.50 a	23.66 ± 0.08 b	0.173 ± 0.009 b	35.37 ± 0.24 d	2.670 ± 0.006 b	6.850 ± 0.023 a
β-carotene 50 mg L⁻¹ × Line-4	53.79 ± 0.74 b	24.32 ± 0.44 a	0.197 ± 0.003 b	48.86 ± 0.66 a	2.360 ± 0.017 c	5.367 ± 0.055 d
β-carotene 75 mg L⁻¹ × Sakha-2	56.52 ± 2.16 b	22.31 ± 0.10 b	0.160 ± 0.000 c	35.13 ± 0.65 d	2.200 ± 0.017 d	6.453 ± 0.153 b
β-carotene 75 mg L⁻¹ × Line-4	61.60 ± 1.43 a	17.41 ± 0.24 c	0.187 ± 0.003 b	46.50 ± 0.75 a	2.103 ± 0.015 d	5.923 ± 0.066 c
Carrot extract 5% × Sakha-2	54.42 ± 2.47 b	21.66 ± 2.14 b	0.183 ± 0.003 b	34.87 ± 0.20 d	2.670 ± 0.006 b	6.363 ± 0.147 b
Carrot extract 5% × Line-4	52.12 ± 2.67 b	21.66 ± 0.86 b	0.167 ± 0.003 c	45.78 ± 0.94 b	2.130 ± 0.017 d	5.207 ± 0.084 e
Carrot extract 10% × Sakha-2	68.32 ± 0.21 a	24.82 ± 1.59 a	0.227 ± 0.003 a	37.15 ± 0.95 d	3.110 ± 0.069 a	7.190 ± 0.133 a
Carrot extract 10% × Line-4	65.57 ± 1.32 a	29.75 ± 0.70 a	0.203 ± 0.003 b	48.38 ± 1.74 a	2.580 ± 0.012 b	5.707 ± 0.020 d
Carrot extract 20% × Sakha-2	63.32 ± 4.36 a	23.55 ± 0.31 b	0.243 ± 0.015 a	36.89 ± 0.40 d	2.680 ± 0.075 b	6.730 ± 0.035 b
Carrot extract 20% × Line-4	53.41 ± 1.09 b	25.63 ± 2.01 a	0.190 ± 0.012 b	46.74 ± 0.77 a	2.307 ± 0.055 c	5.693 ± 0.170 d

Values are means ± SE. Different letters within the same column indicate significant differences according to Tukey’s HSD test at *p* ≤ 0.05

Flavonoid content was highest in Line-4 treated with carrot extract at 10%, reaching 29.75 mg 100 g⁻¹ DW. The highest DPPH radical-scavenging activity was recorded in Line-4 treated with β-carotene at 50 mg L⁻¹, reaching 48.86%, followed closely by Line-4 treated with carrot extract at 10%, which recorded 48.38%. The highest β-carotene content was recorded in Sakha-2 treated with carrot extract at 20%, reaching 0.243, followed by Sakha-2 treated with carrot extract at 10%. These results indicate that antioxidant-related responses were strongly variety-dependent. Sakha-2 showed stronger phenolic, PAL, and TAL responses, whereas Line-4 showed stronger DPPH and flavonoid responses.

### Treatment × variety interaction effects on yield and yield components

The interaction between variety and foliar treatment significantly affected yield and yield-related traits (Tables 10, 11, 12 and 13). Line-4 treated with carrot extract at 20% recorded the highest plant height and fruiting zone length, with values of 105.00 and 31.67 cm, respectively (Table 10). The same interaction also produced the highest number of fruiting branches per plant, reaching 17.67 branches plant⁻¹ (Table 11). In contrast, Line-4 treated with carrot extract at 10% recorded the highest technical stem length, number of capsules per plant, seed yield per plant, seed yield per hectare, and oil yield, with values of 77.33 cm, 68.00 capsules plant⁻¹, 5.44 g plant⁻¹, 1840 kg ha⁻¹, and 598 kg ha⁻¹, respectively (Tables 10 and 11, and 13). These values were higher than those of the untreated Line-4 control, which recorded 50.67 cm technical stem length, 41.00 capsules plant⁻¹, 2.79 g seed yield plant⁻¹, 1030 kg ha⁻¹ seed yield, and 314 kg ha⁻¹ oil yield. Sakha-2 treated with carrot extract at 10% recorded the highest biological yield per plant, reaching 9.27 g plant⁻¹ (Table 12). For field-scale biological yield, the highest value was recorded in Line-4 treated with β-carotene at 50 mg L⁻¹, reaching 10,610 kg ha⁻¹, followed by Sakha-2 treated with carrot extract at 10%, which recorded 10,080 kg ha⁻¹ (Table 13). The highest 1000-seed weight was recorded in Line-4 treated with β-carotene at 75 mg L⁻¹, reaching 6.41 g (Table 12).

**Table 10:** Interaction effect between flax variety and foliar treatment on plant height and stem traits

Treatment	Plant height (cm)	Technical stem length (cm)	Fruiting zone length (cm)
Control × Sakha-2	75.67 ± 0.01 c	57.00 ± 0.51 c	18.67 ± 0.63 c
Control × Line-4	71.67 ± 1.84 c	50.67 ± 0.31 d	21.00 ± 1.30 b
β-carotene 25 mg L⁻¹ × Sakha-2	82.00 ± 1.00 b	61.00 ± 0.72 b	21.00 ± 0.61 b
β-carotene 25 mg L⁻¹ × Line-4	72.33 ± 0.28 c	63.33 ± 0.83 b	9.00 ± 0.02 e
β-carotene 50 mg L⁻¹ × Sakha-2	87.33 ± 2.14 b	64.00 ± 0.83 b	23.33 ± 0.15 b
β-carotene 50 mg L⁻¹ × Line-4	82.67 ± 1.17 b	62.67 ± 0.18 b	20.00 ± 0.62 c
β-carotene 75 mg L⁻¹ × Sakha-2	81.33 ± 0.30 c	58.67 ± 1.27 c	22.67 ± 0.13 b
β-carotene 75 mg L⁻¹ × Line-4	92.00 ± 0.58 b	63.33 ± 0.89 b	28.67 ± 0.65 a
Carrot extract 5% × Sakha-2	85.33 ± 1.50 b	63.33 ± 0.96 b	22.00 ± 0.03 b
Carrot extract 5% × Line-4	89.00 ± 1.33 b	73.00 ± 1.31 a	16.00 ± 0.08 d
Carrot extract 10% × Sakha-2	91.67 ± 1.98 b	67.67 ± 1.76 b	24.00 ± 0.98 b
Carrot extract 10% × Line-4	99.33 ± 5.54 a	77.33 ± 2.40 a	22.00 ± 0.41 b
Carrot extract 20% × Sakha-2	83.33 ± 1.24 b	63.33 ± 2.55 b	20.00 ± 0.10 c
Carrot extract 20% × Line-4	105.00 ± 0.73 a	73.33 ± 1.41 a	31.67 ± 0.25 a

Values are means ± SE. Different letters within the same column indicate significant differences according to Tukey’s HSD test at *p* ≤ 0.05

**Table 11:** Interaction effect between flax variety and foliar treatment on branches, capsules, and plant-level yield traits

Treatment	No. of fruiting branches plant⁻¹	No. of capsules plant⁻¹	Seed yield (g plant⁻¹)
Control × Sakha-2	9.67 ± 0.05 b	23.67 ± 1.07 d	2.960 ± 0.012 b
Control × Line-4	9.67 ± 0.20 b	41.00 ± 0.83 c	2.790 ± 0.144 b
β-carotene 25 mg L⁻¹ × Sakha-2	10.00 ± 0.83 b	36.67 ± 1.44 c	3.290 ± 0.231 b
β-carotene 25 mg L⁻¹ × Line-4	8.67 ± 0.26 c	47.67 ± 3.87 b	2.890 ± 0.162 b
β-carotene 50 mg L⁻¹ × Sakha-2	12.33 ± 0.35 b	52.33 ± 2.40 b	3.660 ± 0.046 b
β-carotene 50 mg L⁻¹ × Line-4	8.33 ± 0.06 c	54.33 ± 3.78 b	3.290 ± 0.006 b
β-carotene 75 mg L⁻¹ × Sakha-2	12.00 ± 0.28 b	39.67 ± 1.20 c	3.400 ± 0.040 b
β-carotene 75 mg L⁻¹ × Line-4	14.67 ± 0.96 a	48.33 ± 1.00 b	3.530 ± 0.052 b
Carrot extract 5% × Sakha-2	13.00 ± 0.40 b	41.33 ± 0.66 c	3.150 ± 0.121 b
Carrot extract 5% × Line-4	6.00 ± 0.08 c	45.00 ± 0.98 c	4.270 ± 0.006 a
Carrot extract 10% × Sakha-2	14.67 ± 0.00 a	56.67 ± 0.44 b	3.910 ± 0.115 b
Carrot extract 10% × Line-4	9.00 ± 0.38 c	68.00 ± 1.68 a	5.440 ± 0.606 a
Carrot extract 20% × Sakha-2	12.67 ± 1.55 b	43.33 ± 1.30 c	3.210 ± 0.133 b
Carrot extract 20% × Line-4	17.67 ± 0.92 a	54.67 ± 3.53 b	5.280 ± 0.346 a

Values are means ± SE. Different letters within the same column indicate significant differences according to Tukey’s HSD test at *p* ≤ 0.05

**Table 12:** Interaction effect between flax variety and foliar treatment on biological yield plant⁻¹, 1000-seed weight, and straw yield

Treatment	Biological yield (g plant⁻¹)	1000-seed weight (g)	Straw yield (kg ha⁻¹)
Control × Sakha-2	4.690 ± 0.035 d	4.250 ± 0.271 b	3480 ± 312 c
Control × Line-4	4.620 ± 0.012 d	4.080 ± 0.208 c	4850 ± 12 c
β-carotene 25 mg L⁻¹ × Sakha-2	6.910 ± 0.012 b	5.270 ± 0.098 b	7680 ± 208 a
β-carotene 25 mg L⁻¹ × Line-4	5.960 ± 0.225 c	5.110 ± 0.046 b	7750 ± 370 a
β-carotene 50 mg L⁻¹ × Sakha-2	8.040 ± 0.191 b	5.620 ± 0.156 a	7897 ± 141 a
β-carotene 50 mg L⁻¹ × Line-4	7.540 ± 0.595 b	6.020 ± 0.231 a	8810 ± 87 a
β-carotene 75 mg L⁻¹ × Sakha-2	7.200 ± 0.075 b	5.070 ± 0.242 b	5807 ± 234 b
β-carotene 75 mg L⁻¹ × Line-4	5.730 ± 0.110 c	6.410 ± 0.075 a	4060 ± 46 c
Carrot extract 5% × Sakha-2	5.790 ± 0.196 c	5.200 ± 0.214 b	4823 ± 43 c
Carrot extract 5% × Line-4	5.140 ± 0.335 c	5.360 ± 0.231 a	4103 ± 107 c
Carrot extract 10% × Sakha-2	9.270 ± 0.294 a	5.750 ± 0.087 a	8423 ± 453 a
Carrot extract 10% × Line-4	7.670 ± 0.110 b	6.300 ± 0.017 a	6407 ± 395 b
Carrot extract 20% × Sakha-2	6.990 ± 0.075 b	5.110 ± 0.139 b	7630 ± 381 a
Carrot extract 20% × Line-4	6.060 ± 0.110 c	6.150 ± 0.370 a	7033 ± 113 b

Values are means ± SE. Different letters within the same column indicate significant differences according to Tukey’s HSD test at *p* ≤ 0.05

**Table 13:** Interaction effect between flax variety and foliar treatment on seed yield, biological yield, oil yield and oil percentage

Treatment	Seed yield (kg ha⁻¹)	Biological yield (kg ha⁻¹)	Oil yield (kg ha⁻¹)	Oil (%)
Control × Sakha-2	860 ± 6 d	4343 ± 372 d	288 ± 6 d	33.57 ± 0.01 b
Control × Line-4	1030 ± 12 d	5880 ± 162 c	314 ± 1 c	30.44 ± 0.18 d
β-carotene 25 mg L⁻¹ × Sakha-2	1140 ± 0 c	8830 ± 421 b	388 ± 13 c	34.07 ± 0.04 a
β-carotene 25 mg L⁻¹ × Line-4	1580 ± 69 b	9313 ± 401 a	502 ± 12 b	31.75 ± 0.11 c
β-carotene 50 mg L⁻¹ × Sakha-2	1643 ± 84 a	9550 ± 381 a	569 ± 37 a	34.66 ± 0.11 a
β-carotene 50 mg L⁻¹ × Line-4	1803 ± 49 a	10,610 ± 289 a	583 ± 11 a	32.34 ± 0.43 c
β-carotene 75 mg L⁻¹ × Sakha-2	1273 ± 9 c	7080 ± 23 c	434 ± 0 b	34.10 ± 0.33 a
β-carotene 75 mg L⁻¹ × Line-4	1320 ± 52 c	5377 ± 124 d	426 ± 4 b	32.30 ± 0.06 c
Carrot extract 5% × Sakha-2	1337 ± 3 c	6167 ± 182 c	449 ± 23 b	33.57 ± 0.21 b
Carrot extract 5% × Line-4	1633 ± 32 a	5737 ± 153 c	497 ± 5 b	30.44 ± 0.36 d
Carrot extract 10% × Sakha-2	1657 ± 3 a	10,080 ± 352 a	569 ± 15 a	34.34 ± 0.32 a
Carrot extract 10% × Line-4	1840 ± 87 a	8233 ± 170 b	598 ± 1 a	32.52 ± 0.23 b
Carrot extract 20% × Sakha-2	1297 ± 55 c	8930 ± 237 b	454 ± 15 b	34.98 ± 0.17 a
Carrot extract 20% × Line-4	1580 ± 17 b	8620 ± 17 b	522 ± 28 a	33.06 ± 0.10 b

Values are means ± SE. Different letters within the same column indicate significant differences according to Tukey’s HSD test at *p* ≤ 0.05

### Integrated trait relationships and multivariate response patterns

**Fig. 1:**
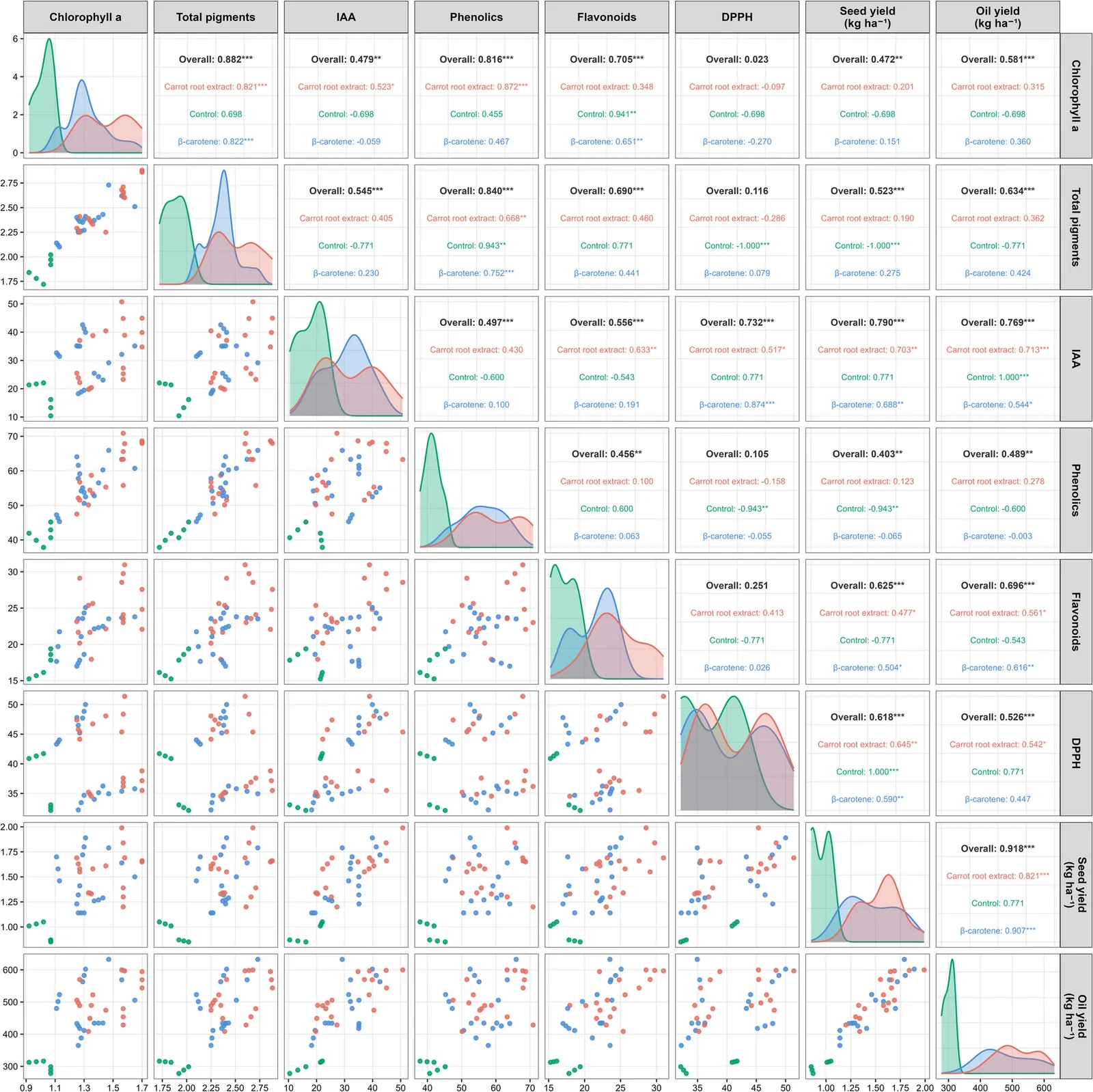
Scatterplot matrix showing pairwise relationships among selected physiological, biochemical, and yield-related traits of flax. The lower panels show scatterplots of the original replicate-level observations, the diagonal panels show density distributions, and the upper panels show Spearman correlation coefficients for the complete dataset and within each foliar-treatment category. The overall correlations were calculated using all original replicate-level observations (*n* = 42; two varieties × seven foliar treatments × three replicates). The treatment-category-specific correlations were calculated using the corresponding original observations for the control (*n* = 6), β-carotene treatments (*n* = 18), and carrot root extract treatments (*n* = 18). Colors distinguish the control, β-carotene, and carrot root extract categories. *, **, and *** indicate significance at *P* < 0.05, *P* < 0.01, and *P* < 0.001, respectively

The scatterplot matrix of selected traits was used as an exploratory visualization to confirm pairwise relationships among key physiological and yield traits (Fig. 1). The matrix showed clear positive associations among chlorophyll a, total pigments, phenolics, flavonoids, IAA, seed yield, and oil yield. These patterns further support the interpretation that improved flax productivity under the most effective foliar treatments was linked with enhanced pigment accumulation, antioxidant-related metabolism, and reproductive yield formation.

**Fig. 2:**
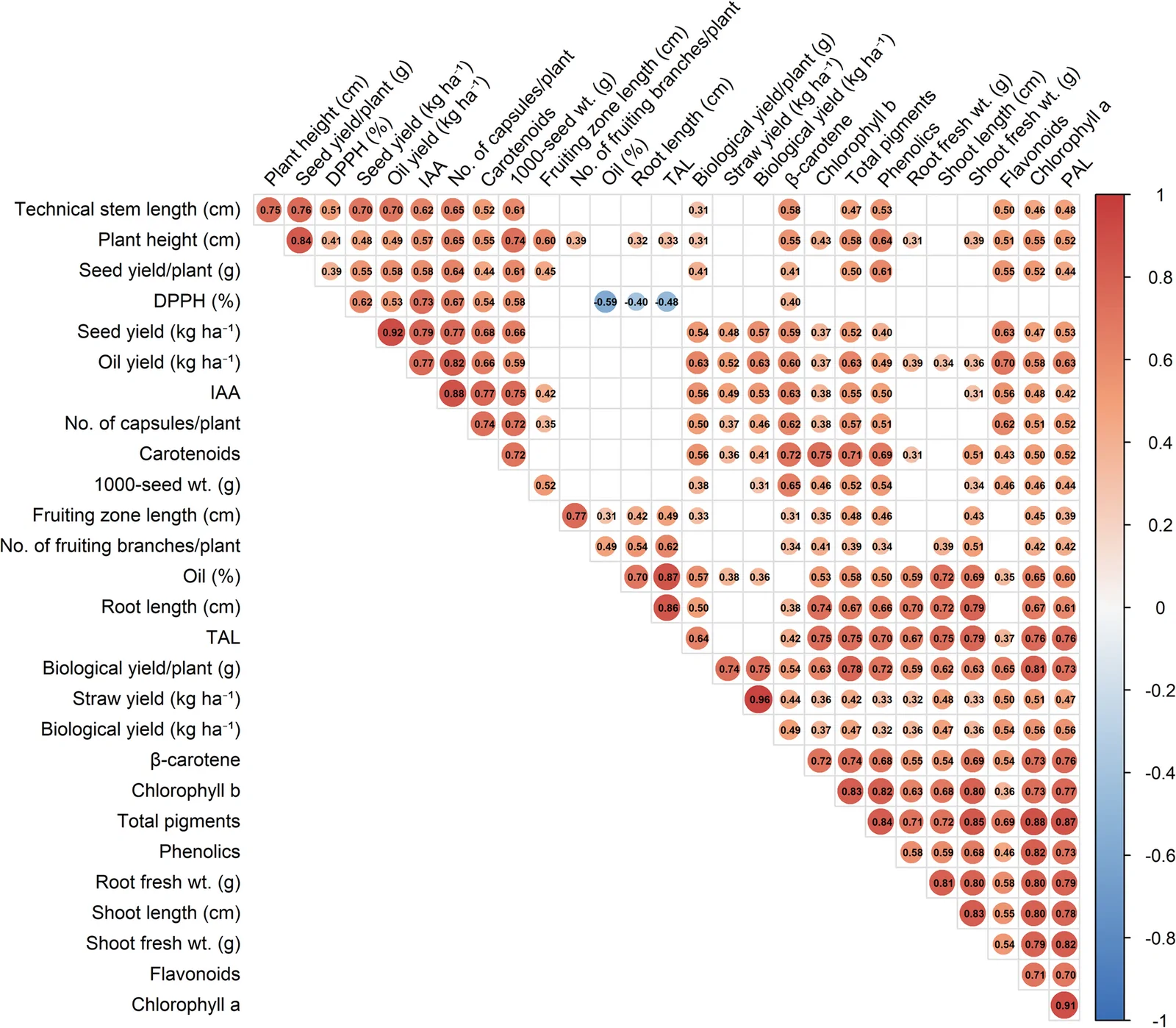
Spearman correlation plot showing statistically significant relationships among growth, physiological, biochemical, antioxidant-related, and yield traits of flax. Spearman correlation coefficients were calculated using the original replicate-level observations (*n* = 42; two varieties × seven foliar treatments × three replicates), without averaging the observations by treatment. Only correlations significant at *P* ≤ 0.05 are displayed. Circle size and color intensity represent the magnitude and direction of Spearman’s correlation coefficient (ρ), while the numerical values inside the circles indicate the corresponding correlation coefficients. Red and blue circles indicate positive and negative correlations, respectively

Spearman correlation analysis was performed to explore the relationships among growth, physiological, biochemical, antioxidant-related, and yield traits using the original replicate-level dataset (Fig. 2). The analysis revealed strong positive associations among several yield-related traits. Seed yield was highly correlated with oil yield, seed yield per plant, number of capsules per plant, technical stem length, and plant height. Oil yield also showed positive correlations with seed yield, IAA, flavonoids, chlorophyll a, and PAL activity, indicating that yield improvement was associated not only with reproductive traits but also with enhanced physiological and biochemical performance.

Photosynthetic and antioxidant-related traits were also closely connected. Chlorophyll a showed a strong positive correlation with total pigments, phenolics, flavonoids, β-carotene, and PAL activity. Total pigments were positively correlated with chlorophyll b, phenolics, β-carotene, shoot length, shoot fresh weight, and PAL activity. These relationships suggest that treatments improving pigment accumulation also tended to enhance antioxidant-related metabolism and vegetative growth. In addition, PAL and TAL activities were positively associated with phenolics, flavonoids, β-carotene, root growth, shoot growth, and oil yield, supporting the involvement of phenylpropanoid-related metabolism in the overall flax response to foliar β-carotene and carrot root extract.

**Fig. 3:**
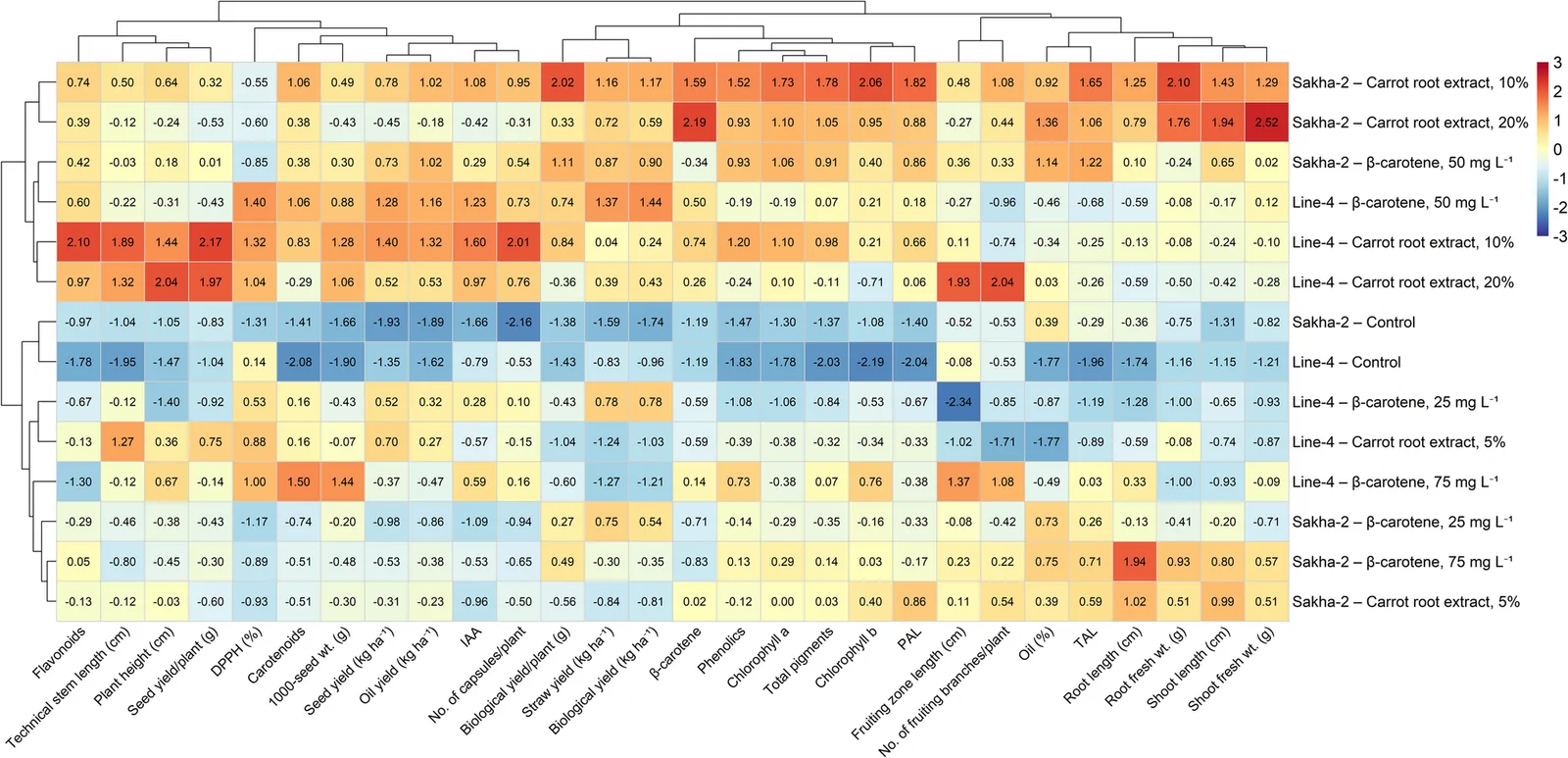
Clustered heatmap showing Z-score-standardized treatment means for growth, physiological, biochemical, antioxidant-related, and yield traits of flax. Z-scores were calculated separately for each trait across the treatment combinations. Positive and negative Z-scores indicate values above and below the overall mean of the corresponding trait, respectively. Hierarchical clustering was applied to both treatment combinations and traits. The heatmap was used solely as a descriptive visualization and was not used for correlation calculations or inferential statistical analysis

The clustered heatmap provided a complementary overview of treatment response patterns (Fig. 3). The untreated control treatments of both varieties formed a low-response cluster, showing negative Z-scores for most growth, physiological, biochemical, and yield traits. In contrast, carrot extract at 10% was clearly separated from the controls and showed high positive Z-scores for several key traits, particularly seed yield, oil yield, IAA, total pigments, chlorophyll a, phenolics, flavonoids, PAL, TAL, and root and shoot growth traits. Sakha-2 treated with carrot extract at 10% showed a strong physiological and biochemical response, whereas Line-4 treated with carrot extract at 10% showed the strongest reproductive and yield-related response. These results confirm that carrot extract at 10% was the most balanced treatment across physiological, biochemical, and yield traits.

## Discussion

The present study demonstrated that foliar application of β-carotene and carrot root extract improved the growth, physiological status, antioxidant-related traits, and yield performance of flax grown in sandy soil during a field-imposed short-term water-deficit episode. These findings support the hypothesis that exogenous antioxidant-related treatments can sustain plant performance during periods of limited water availability. The magnitude of the response varied according to treatment, concentration, genotype, and measured trait, indicating that the effects were both treatment- and trait-specific. The coordinated improvements in physiological, biochemical, and yield-related variables are consistent with recent flax studies in which foliar stress-mitigation treatments enhanced productivity, antioxidant defense, photosynthetic performance, and stress-related metabolism under water-deficit or salinity conditions [3–5].

The improvement in plant growth under β-carotene and carrot extract treatments may be attributed to better physiological stability during water limitation. Sandy soils generally have low water-holding capacity, which can rapidly expose plants to transient water deficit and reduce cell expansion, biomass accumulation, and assimilate production. In the present study, the increase in shoot length and shoot fresh weight, especially under carrot extract at 10% and 20%, suggest that treated plants maintained stronger vegetative growth than untreated plants. Similar responses have been reported in flax under abiotic-stress mitigation treatments, where foliar thiourea improved growth under salt stress by reducing oxidative injury and improving antioxidant defense and nutrient balance [10]. Foliar selenium application in flax also improved capsule number, seed yield, oil yield, and antioxidant-related traits, supporting the concept that exogenous stress-mitigation treatments can strengthen physiological performance and yield formation in flax [4].

The increases in chlorophyll a and total pigment contents were associated with better maintenance of the photosynthetic apparatus during the field-imposed short-term water-deficit episode. Water deficit commonly disturbs chlorophyll biosynthesis, accelerates pigment degradation, and restricts photosynthetic electron transport, which ultimately reduces assimilate supply for growth and seed development. Carotenoids play a dual role in photosynthesis because they contribute to light harvesting and photoprotection by dissipating excess energy and limiting photooxidative damage. Therefore, the positive effect of β-carotene at 50 mg L⁻¹ may be partly explained by its role as a carotenoid-associated antioxidant and photoprotective compound. Previous work showed that exogenous β-carotene can reduce oxidative injury, improve chlorophyll retention, and enhance antioxidant activity under abiotic stress, supporting its potential role in maintaining photosynthetic function [18].

Carrot root extract, particularly at 10%, produced broader improvements than β-carotene alone across growth, physiological, biochemical, and yield-related traits. This response likely reflects the combined action of its naturally occurring constituents, including carotenoids, phenolic compounds, vitamins, tocopherols, and other redox-active metabolites, rather than carotenoid supply alone. Previous studies have reported that carrot-derived extracts can enhance antioxidant responses and seed viability under salinity, while carotenoids, phenolics, and antioxidant-related metabolism in carrot contribute to stress adaptation [14, 21]. Collectively, these findings support the potential role of carrot root extract as a natural multi-component biostimulant in flax. Because comprehensive compound-level phytochemical profiling was not performed, the observed responses are most appropriately attributed to the whole extract rather than to any single constituent.

The marked increase in IAA following carrot root extract application provides a plausible physiological explanation for part of the observed growth response. Auxin plays a central role in cell division, cell elongation, vascular differentiation, and root–shoot coordination, all of which contribute to maintaining plant growth under limited water availability. The concurrent increases in IAA, shoot growth, and yield-related traits support the involvement of auxin-related growth regulation in the response to carrot root extract [22]. Together with the improvements in photosynthetic pigments and antioxidant-related traits, this response may have contributed to the superior performance of plants treated with carrot root extract at 10%.

The increases in phenolic and flavonoid contents, together with higher DPPH radical-scavenging activity, were associated with enhanced non-enzymatic antioxidant capacity in plants treated with β-carotene and carrot root extract. Phenolics and flavonoids are important secondary metabolites that may contribute to reactive oxygen species scavenging, metal-ion chelation, and the protection of cellular membranes and photosynthetic tissues from oxidative injury. In flax, phenolic profiles and antioxidant capacity vary with growth stage and contribute to the plant’s redox potential and stress-response capacity [22]. Previous research also showed that foliar application of esculin and digitoxin increased phenolic accumulation and antioxidant defense in salt-stressed flax, in association with improved growth and yield quality [5]. Therefore, the concurrent increases in phenolics, flavonoids, DPPH radical-scavenging activity, growth, and productivity observed in the present study suggest a possible contribution of non-enzymatic antioxidant processes to the responses of treated plants.

The increases in PAL and TAL activities were consistent with the possible involvement of phenylpropanoid metabolism in the response to foliar treatments. PAL is a key entry-point enzyme in phenylpropanoid biosynthesis, linking primary metabolism to the formation of phenolics, flavonoids, lignin precursors, and other defense-related compounds. TAL also contributes to phenolic metabolism through the conversion of tyrosine-derived substrates into phenylpropanoid intermediates. The higher activities of these enzymes following carrot root extract and β-carotene application may indicate enhanced phenylpropanoid-related metabolism associated with antioxidant protection and structural defense. However, because the activities of downstream enzymes and the concentrations of individual phenylpropanoid metabolites were not determined, the present results should be interpreted as evidence of an association rather than direct confirmation of pathway activation. Similar associations were reported in salt-stressed flax, where improvements in growth and yield quality coincided with higher PAL and TAL activities and enhanced antioxidant defense [5].

The higher yields obtained with carrot root extract at 10% and β-carotene at 50 mg L⁻¹ were accompanied by improvements in several physiological and biochemical traits. Increases in seed yield, biological yield, 1000-seed weight, oil percentage, and oil yield show that the benefits of these treatments extended beyond vegetative growth to reproductive development and economically important yield outcomes.

Seed and oil yields in flax are highly sensitive to limited water availability during flowering and seed filling, when impaired photosynthetic activity and oxidative stress can restrict capsule formation, seed development, and oil accumulation. Therefore, the improved pigment status and antioxidant-related metabolism observed in treated plants likely supported reproductive performance and yield formation. This interpretation agrees with recent studies showing that foliar stress-mitigation treatments improved yield and quality while enhancing antioxidant defense and reducing oxidative damage in flax [3, 4].

The response to carrot root extract was concentration- and trait-dependent rather than strictly linear. Although 20% produced the highest values for some growth traits, 10% resulted in more consistent improvements across photosynthetic pigments, IAA concentration, phenolic and flavonoid contents, antioxidant-related enzyme activities, seed yield, and oil yield. This pattern indicates that increasing the extract concentration beyond 10% did not uniformly enhance all measured traits and that the most effective concentration varied according to the response variable. Under the present field conditions, 10% provided the most balanced overall response, whereas 20% remained advantageous for selected growth traits. Further dose–response studies would help refine concentration-specific recommendations for flax.

The varietal differences observed between Sakha-2 and Line-4 indicate that the response to foliar antioxidant-based treatments was genotype-dependent. Such variation may reflect differences in baseline growth capacity, pigment stability, antioxidant metabolism, phenolic accumulation, and reproductive sink strength. Genotype-dependent responses to drought and foliar treatments have been reported in flax and other crops, indicating that the effectiveness of stress-mitigation treatments can depend strongly on the genetic background of the plant. This is important for practical recommendation because the same foliar treatment may not produce identical benefits across all flax varieties. Therefore, the treatment × variety interaction observed in the present study supports the need to evaluate foliar biostimulants across diverse genotypes before making broad agronomic recommendations.

Overall, foliar application of β-carotene and carrot root extract induced coordinated improvements in photosynthetic pigments, IAA concentration, phenylpropanoid-related enzyme activities, antioxidant-related traits, and yield formation. The novelty of the present study lies in the direct comparison of a purified carotenoid treatment with a natural carrot-derived extract in two flax varieties grown in sandy soil during a field-imposed short-term water-deficit episode. β-Carotene at 50 mg L⁻¹ produced marked yield improvements, whereas carrot root extract at 10% generated the broadest and most balanced response across physiological, biochemical, and productivity traits. These findings demonstrate the potential of both treatments as foliar strategies for improving flax performance and highlight the particularly strong integrated response achieved with 10% carrot root extract.

These findings should be interpreted as responses to a field-imposed short-term water-deficit episode under sprinkler-irrigation conditions. Although the foliar treatments improved growth, photosynthetic pigments, antioxidant-related traits, and yield, additional research incorporating direct soil-moisture monitoring, plant water-status measurements, gas exchange, chlorophyll fluorescence, oxidative-stress indicators, and detailed phytochemical profiling of carrot root extract would provide further insight into the physiological basis of these responses and support their validation across different environments and irrigation regimes.

## Materials & methods

### Experimental site and soil conditions

Two field experiments were carried out during the 2021/2022 and 2022/2023 winter seasons at the Experimental Station of the National Research Centre, Al-Nubaria district, El-Beheira Governorate, Egypt. The experimental site is located at approximately 30°30′1.4″ N latitude, 30°19′10.9″ E longitude, and 21 m above mean sea level. The soil of the experimental site was sandy. Before sowing, representative soil samples were collected from the experimental area to determine the initial mechanical, chemical, and nutritional properties of the soil. Soil analyses were carried out according to Chapman and Pratt [23], and the main soil characteristics are presented in Tables 14, 15 and 16.

**Table 14:** Initial mechanical properties of the experimental soil

Treatment	Coarse sand 2000–200 µ (%)	Fine sand 200–20 µ (%)	Silt 20–0 µ (%)	Clay <2 µ (%)	Soil texture
Soil	47.460 ± 0.548	36.190 ± 0.418	12.860 ± 0.148	4.280 ± 0.049	Sandy

**Table 15:** Initial chemical properties of the experimental soil

Treatment	pH (1:2.5)	EC (dS m-1)	CaCO3 (%)	OM (%)	Na+ (meq/l)	K+ (meq/l)
Soil	7.600 ± 0.088	0.130 ± 0.002	5.300 ± 0.061	0.060 ± 0.001	0.570 ± 0.007	0.130 ± 0.002

Treatment	Mg2+ (meq/l)	Ca2+ (meq/l)	CO3 2- (meq/l)	HCO3- (meq/l)	Cl- (meq/l)	SO4 2- (meq/l)
Soil	0.920 ± 0.011	1.00 ± 0.01	0.000 ± 0.000	1.250 ± 0.014	0.480 ± 0.006	0.890 ± 0.010

**Table 16:** Initial nutritional properties of the experimental soil

Treatment	Available *N* (ppm)	Available *P* (ppm)	Available K (ppm)	Zn (ppm)	Fe (ppm)	Mn (ppm)	Cu (ppm)
Soil	52.00 ± 0.60	12.00 ± 0.14	75.00 ± 0.87	0.140 ± 0.002	1.400 ± 0.016	0.300 ± 0.003	0.000 ± 0.000

### Plant material and chemical sources

Certified seeds of two flax (*Linum usitatissimum* L.) varieties, Sakha-2 and Line-4, were obtained from the Agricultural Research Center, Egypt. Fresh carrot (*Daucus carota* L.) roots were purchased from local markets in Egypt and used to prepare carrot root extract. The plant materials used in this study consisted exclusively of cultivated varieties. All experimental procedures were conducted in accordance with relevant institutional and national guidelines for agricultural research. All chemicals and reagents used in the biochemical analyses were of analytical grade.

### Experimental design and treatment structure

The experiment was arranged in a split-plot design with three replications. The two flax varieties, Sakha-2 and Line-4, were assigned to the main plots, whereas foliar treatments were randomly allocated to the subplots. Seven foliar treatments were evaluated: untreated control, β-carotene at 25, 50, and 75 mg L⁻¹, and carrot root extract at 5, 10, and 20% (v/v). The control was a common untreated control for both foliar-treatment series and was sprayed with water only at the same application times.

The β-carotene concentrations used in the present study, 25, 50, and 75 mg L⁻¹, correspond approximately to 0.047, 0.093, and 0.140 mM, respectively. These concentrations were selected to represent a low foliar range compared with previously reported exogenous β-carotene applications in plants under stress conditions. Babaei et al. [18] evaluated β-carotene concentrations of 0, 0.5, 2.5, and 5 mM during preliminary screening and selected 0.5 mM for the main experiment, reporting improved chlorophyll content, antioxidant activity, phenolic accumulation, and oxidative-stress mitigation in salt-stressed *Lepidium sativum*. El-Adl et al. [24] also applied β-carotene to salt-stressed squash after screening a range of concentrations from 0 to 300 ppm. Therefore, the concentrations used in the present study were selected as conservative low-dose foliar treatments suitable for field evaluation in flax. The carrot root extract concentrations, 5, 10, and 20%, were selected as graded extract dilutions to evaluate low, moderate, and high levels of a natural multi-component biostimulant.

### Preparation of carrot root extract

Carrot root extract was prepared according to Sofowora [25], with minor adaptation for foliar application. Fresh carrot roots were washed thoroughly with distilled water, cut into small pieces, and homogenized using an electric blender. Briefly, 100 g of washed fresh carrot roots were blended with 160 mL sterile distilled water and 160 mL ethanol. The homogenate was transferred to a flask, shaken for 1 h at room temperature, and filtered through Whatman No. 1 filter paper. The pH of the filtrate was adjusted to 7.0 using 1 N NaOH, and the final volume was completed with sterile distilled water. The prepared extract was diluted with distilled water to obtain the final foliar concentrations of 5, 10, and 20% (v/v). The extract was freshly prepared before foliar application to reduce possible degradation of bioactive constituents. The vitamin, mineral, and proximate compositions of the carrot root extract were determined experimentally using standard analytical procedures and are presented in Tables 17, 18 and 19. However, comprehensive compound-level phytochemical profiling was not performed; therefore, the identities and concentrations of individual phenolic acids, flavonoids, and other specialized metabolites in the extract remain unknown.

**Table 17:** Vitamin composition of carrot root extract used for foliar application

Treatment	Vit. A (as β-carotene)	Vit C	Vit B1	Vit B6	Vit B2	Vit E	Folic acid
Carrot roots extract	11.00 ± 0.13	35.00 ± 0.40	1.50 ± 0.02	1.700 ± 0.020	2.00 ± 0.02	2.100 ± 0.024	1.360 ± 0.016

**Table 18:** Mineral composition of carrot root extract used for foliar application

Treatment	Potassium	Calcium	Magnesium	Phosphorus	Sulphur	Copper	Iron
Carrot roots extract	63.50 ± 0.73	69.00 ± 0.80	2.00 ± 0.02	1.450 ± 0.017	0.920 ± 0.011	0.800 ± 0.009	1.300 ± 0.015

**Table 19:** Other chemical constituents of carrot root extract used for foliar application

Treatment	Carbohydrates (%)	Protein (%)	Fat (%)	Essential oil (%)
Carrot roots extract	18.00 ± 0.21	12.00 ± 0.14	1.50 ± 0.02	0.590 ± 0.007

### Preparation of β-carotene solutions and application of foliar treatments

β-Carotene foliar solutions were prepared according to the approach described by El-Adl et al. [24], with modifications to obtain the concentrations used in the present study. Briefly, β-carotene was first dissolved in methanol to prepare a stock solution, which was then diluted with distilled water adjusted to pH 6.0 to obtain the final working concentrations of 25, 50, and 75 mg L⁻¹. The working solutions were freshly prepared before each foliar application.

Foliar applications of β-carotene and carrot root extract were carried out twice during the growing season, at 30 and 45 days after sowing. β-Carotene was applied at 25, 50, and 75 mg L⁻¹, while carrot root extract was applied at 5, 10, and 20% (v/v). Spraying was performed until complete leaf wetting. Untreated control plants were sprayed with water at the same application times. All foliar treatments were applied under similar field conditions using the same spraying procedure to ensure uniform application among treatments.

### Agronomic practices

Flax seeds of Sakha-2 and Line-4 were sown on 1 November in both growing seasons. Seeds were drilled in rows 3.5 m long, with 20 cm spacing between rows. The subplot area was 10.5 m², measuring 3.0 m × 3.5 m. The seeding rate was 2000 seeds m⁻². Before sowing, calcium superphosphate fertilizer, 15.5% P₂O₅, was applied at 360 kg ha⁻¹ during seedbed preparation. Nitrogen fertilizer was applied after emergence in the form of ammonium nitrate, 33.5% N, at 180 kg ha⁻¹, divided into five equal doses. Potassium sulfate, 48% K₂O, was applied at 120 kg ha⁻¹ in two equal doses. Other agronomic practices recommended for flax production in the experimental region were applied uniformly to all treatments.

### Irrigation management and field-imposed water-deficit episode

Irrigation was applied through a sprinkler irrigation system at fixed 5-day intervals throughout the growing season, except during the imposed water-deficit treatment. The irrigation amount for each interval was estimated from Class A pan evaporation using the FAO-56 crop-coefficient approach according to Allen et al., [26]:

$$\mathrm{ETo}=\mathrm{Epan}\times \mathrm{Kp}$$

$$\mathrm{ETc}=\mathrm{ETo}\times \mathrm{Kc}$$

$$\mathrm{IWR}=\mathrm{ETc}\times10\times1\mathrm{.}2$$

where ETo is the reference evapotranspiration, Epan is the pan evaporation, Kp is the pan coefficient, ETc is the crop evapotranspiration for the relevant irrigation interval, Kc is the crop coefficient, and IWR is the irrigation water requirement. ETo and ETc were expressed in mm, whereas IWR was expressed in m³ ha⁻¹; the factor 10 converts a water depth of 1 mm over 1 ha to 10 m³ ha⁻¹, and 1.2 represents the leaching allowance adopted for sandy-soil conditions.

A short-term water-deficit episode was imposed by withholding one scheduled irrigation immediately after the second foliar spray, which was applied at 45 days after sowing (DAS). Under the routine 5-day irrigation schedule, this corresponded to one omitted irrigation cycle, equivalent to approximately five days without irrigation, after which irrigation was resumed according to the regular schedule. Because direct measurements of soil moisture, field capacity, relative water content, leaf water potential, or other plant water-status indicators were not recorded, the treatment was interpreted as a field-imposed short-term water-deficit episode rather than as a fully quantified drought-stress treatment.

In sandy soils, omission of a single scheduled irrigation may produce a biologically meaningful transient decline in plant-available water because these soils have low water-holding capacity and require frequent irrigation to meet atmospheric demand. This consideration is particularly relevant for flax because its relatively shallow root system may increase its sensitivity to short periods of limited water availability. However, because soil moisture and plant water-status indicators were not measured directly, the treatment effects are interpreted as responses to a field-imposed short-term water-deficit episode rather than to a quantitatively defined level of drought stress [27–29].

### Sampling procedures and measured traits

Plant samples were collected at 60 days after sowing to determine growth traits and biochemical parameters. Representative plants were sampled from each subplot. Growth traits included shoot length (cm), shoot fresh weight (g), root length (cm), and root fresh weight (g). Fresh leaf samples were used for determining photosynthetic pigments, endogenous indole-3-acetic acid, total phenolics, flavonoids, β-carotene, DPPH radical-scavenging activity, and PAL and TAL enzyme activities.

At maturity, flax plants from each whole plot were pulled when signs of full maturity appeared and then left on the ground until completely dry. Capsules were removed carefully. Ten individual plants were taken from each plot to estimate plant height (cm), technical stem length (cm), fruiting zone length (cm), number of fruiting branches per plant, number of capsules per plant, seed yield per plant (g), biological yield per plant (g), and 1000-seed weight (g). Straw yield, seed yield, and biological yield were calculated for each treatment and expressed as kg ha⁻¹. Oil yield was calculated from seed yield and seed oil percentage according to the following equation:

$$\mathrm{Oil}\,\,\mathrm{yield}\,(\mathrm{kg}\,\mathrm{ha}^{-1})=\mathrm{see}\,\mathrm{yield}\,(\mathrm{kg}\,\mathrm{ha}^{-1})\times\mathrm{seed}\,\mathrm{oil}\,\mathrm{percentage}/100$$

### Chemical and biochemical analyses

#### Determination of photosynthetic pigments

Photosynthetic pigments, including chlorophyll a, chlorophyll b, and total carotenoids, were determined in fresh flax leaves using a spectrophotometric method based on the equations described by Chazaux et al. [30]. Fresh leaf tissue was ground in 80% acetone using a mortar and pestle. The extract was clarified, and absorbance was measured spectrophotometrically at the appropriate wavelengths. Chlorophyll a, chlorophyll b, carotenoids, and total pigments were calculated and expressed as mg g⁻¹ fresh weight.

#### Determination of endogenous indole-3-acetic acid

Endogenous indole-3-acetic acid (IAA) was determined according to Gusmiaty et al. [31]. A known weight of fresh leaf tissue was extracted three times with 85% cold methanol at 0 °C. The combined methanolic extracts were collected and brought to a known volume with cold methanol. One milliliter of the methanolic extract was mixed with 4 mL of para-dimethylaminobenzaldehyde reagent. The reagent was prepared by dissolving 1 g of para-dimethylaminobenzaldehyde in 50 mL of HCl and 50 mL of ethanol. The mixture was incubated for 60 min at 30–40 °C, and the developed color was measured spectrophotometrically at 530 nm. IAA concentration was calculated using a standard curve and expressed according to the units shown in the tables.

#### Determination of total phenolic content

Total phenolic content was determined in fresh flax leaves using the Folin–Ciocalteu reagent according to Ainsworth and Gillespie [32]. One gram of fresh leaf tissue was extracted three times with 50 mL of 85% cold methanol. The combined extracts were collected and made up to a known volume with cold methanol. One milliliter of the extract was mixed with 0.5 mL of Folin–Ciocalteu reagent and allowed to stand for 3 min. Then, 2 mL of saturated sodium carbonate solution was added, followed by distilled water. The mixture was shaken and left for 60 min. Absorbance was measured at 750 nm using a spectrophotometer. Total phenolic content was calculated from a standard curve and expressed according to the units shown in the tables.

#### Determination of flavonoid content

Total flavonoid content was determined according to Chang et al. [33] using the aluminum chloride colorimetric method. Briefly, 50 µL of crude extract was adjusted to 1 mL with methanol, mixed with 4 mL of distilled water, and then 0.3 mL of 5% NaNO₂ solution was added. After 5 min, 0.3 mL of 10% AlCl₃ solution was added, and the mixture was allowed to stand for 6 min. Then, 2 mL of 1 M NaOH solution was added, and the final volume was brought to 10 mL with double-distilled water. The mixture was incubated for 15 min, and absorbance was measured at 510 nm. Flavonoid content was calculated from a rutin calibration curve and expressed according to the units shown in the tables.

#### Determination of PAL and TAL activities

Phenylalanine ammonia-lyase (PAL) and tyrosine ammonia-lyase (TAL) activities were determined according to Haard and Wasserman [34]. A 0.5-g sample of fresh leaf tissue was homogenized in 3 mL of ice-cold sodium borate buffer, pH 8.8, containing 1.4 mM of 2-mercaptoethanol and 0.1 g of polyvinylpyrrolidone. The homogenate was centrifuged at 10,000 rpm for 25 min, and the supernatant was used for enzyme assays.

PAL activity was determined by mixing 0.1 mL of enzyme extract with 0.1 M of trisodium borate buffer, pH 8.5, and 0.5 mL of 12 mM L-phenylalanine. The final volume was brought to 3 mL with deionized water, and the reaction mixture was incubated at 30 °C for 30 min. The increase in absorbance was recorded at 270 nm for 5 min. TAL activity was determined by measuring the formation of p-coumaric acid at 333 nm. Enzyme activities were calculated and expressed according to the units shown in the tables.

#### Determination of seed oil percentage

Seed oil percentage was determined using a Soxhlet extraction apparatus according to Das et al. [35]. Dried flaxseed samples were ground into a fine powder. A known weight of powdered seed material was placed in a Soxhlet extraction apparatus and extracted using an organic solvent until complete oil extraction. After extraction, the solvent was evaporated, and the extracted oil was dried and weighed. Seed oil percentage was calculated on a seed dry-weight basis using the following equation:

$$\begin{aligned}&\mathrm{O}\mathrm{i}\mathrm{l}\:\text({\%})\:=\:\mathrm{e}\mathrm{x}\mathrm{t}\mathrm{r}\mathrm{a}\mathrm{c}\mathrm{t}\mathrm{e}\mathrm{d}\:\mathrm{o}\mathrm{i}\mathrm{l}\:\mathrm{w}\mathrm{e}\mathrm{i}\mathrm{g}\mathrm{h}\mathrm{t}/\\&\mathrm{d}\mathrm{r}\mathrm{y}\:\mathrm{s}\mathrm{e}\mathrm{e}\mathrm{d}\:\mathrm{s}\mathrm{a}\mathrm{m}\mathrm{p}\mathrm{l}\mathrm{e}\:\mathrm{w}\mathrm{e}\mathrm{i}\mathrm{g}\mathrm{h}\mathrm{t}\:\times\:\:100\end{aligned}$$

#### Determination of DPPH radical-scavenging activity

Free radical-scavenging activity was determined using the DPPH assay according to Gyamfi et al. [36]. Briefly, 50 µL of methanolic plant extract, yielding 100 µg mL⁻¹ in the reaction mixture, was mixed with 1 mL of 0.1 mM DPPH solution in methanol and 450 µL of 50 mM Tris-HCl buffer, pH 7.4. Methanol was used as the blank. The reaction mixture was incubated for 30 min at room temperature, and absorbance was measured at 517 nm. L-ascorbic acid and butylated hydroxytoluene were used as reference antioxidants. DPPH inhibition percentage was calculated using the following equation:

$$\begin{aligned}\:\mathrm{D}\mathrm{P}\mathrm{P}\mathrm{H}&\:\mathrm{i}\mathrm{n}\mathrm{h}\mathrm{i}\mathrm{b}\mathrm{i}\mathrm{t}\mathrm{i}\mathrm{o}\mathrm{n}\;(\%)\:=\:\left[\right(\mathrm{A}\mathrm{c}\mathrm{o}\mathrm{n}\mathrm{t}\mathrm{r}\mathrm{o}\mathrm{l}\hspace{0.17em}-\\&\hspace{0.17em}\mathrm{A}\mathrm{s}\mathrm{a}\mathrm{m}\mathrm{p}\mathrm{l}\mathrm{e})\:/\:\mathrm{A}\mathrm{c}\mathrm{o}\mathrm{n}\mathrm{t}\mathrm{r}\mathrm{o}\mathrm{l}]\:\times\:\:100\end{aligned}$$

where Acontrol is the absorbance of the control reaction and Asample is the absorbance of the tested sample.

#### Determination of β-carotene content

β-Carotene content in flax leaves was determined according to Nagata and Yamashita [37]. One gram of ground flax leaf tissue was extracted with acetone/hexane, 4:6 v/v. The extract was homogenized thoroughly and clarified before spectrophotometric measurement. Absorbance was recorded at 453, 505, 645, and 663 nm using a UV–visible spectrophotometer. β-Carotene content was calculated using the following equation:

$$\begin{aligned} \beta-\mathrm{carotene}\, &(\mathrm{mg}\,100\mathrm{mL}^{-1})=0\mathrm{.}216\mathrm{A}663-\\&1\mathrm{.}22\mathrm{A}645-0\mathrm{.}304\mathrm{A}505+0\mathrm{.}452\mathrm{A}453\end{aligned}$$

The obtained values were then converted and expressed on a leaf dry-weight basis, as shown in the tables.

### Statistical analysis

The original replicate-level dataset was analyzed using R software. Data from the two growing seasons were subjected to combined analysis after testing the homogeneity of error variances. Season was considered as a random factor, whereas variety and foliar treatment were considered fixed factors. All inferential statistical analyses were conducted using the original replicate-level observations. For the multivariate analyses, the scatterplot matrix and Spearman correlation plot were generated using the original replicate-level observations (*n* = 42; two varieties × seven foliar treatments × three replicates). In contrast, the clustered heatmap was constructed using treatment means transformed into Z-scores separately for each trait. The heatmap was used as a descriptive visualization of relative treatment-response patterns and was not used for correlation calculations or inferential statistical analysis. The experiment was analyzed according to a split-plot design with three replications. Flax variety was considered the main-plot factor, and foliar treatment was considered the subplot factor. Analysis of variance was performed to test the effects of variety, foliar treatment, and their interaction. Residual diagnostics were examined to verify the suitability of the ANOVA model before mean separation. When significant differences were detected, treatment means were compared using Tukey’s honestly significant difference test at *P* ≤ 0.05. Results are presented as means ± standard error (SE), and different letters indicate significant differences among means.

### Integrated correlation and multivariate visualization analyses

To provide an integrated interpretation of the relationships among growth, physiological, biochemical, antioxidant-related, and yield traits, additional correlation and multivariate visualization analyses were conducted using R statistical software [38]. Both the scatterplot matrix and the Spearman correlation analysis were based exclusively on the original replicate-level observations (*n* = 42); treatment means were not used to calculate the reported correlation coefficients.

A scatterplot matrix was generated to examine pairwise relationships among selected key physiological, biochemical, and yield-related traits. The selected traits included chlorophyll a, total pigments, IAA, phenolic and flavonoid contents, DPPH radical-scavenging activity, seed yield, and oil yield. The scatterplot matrix was generated using the original replicate-level observations (*n* = 42; two varieties × seven foliar treatments × three replicates), without averaging the observations by treatment. Lower panels show pairwise scatterplots, diagonal panels show density distributions, and upper panels show Spearman correlation coefficients. The scatterplot matrix was generated using the GGally package in R, together with ggplot2 for graphical formatting [39, 40].

Spearman correlation analysis was performed using the original replicate-level observations (*n* = 42), representing two flax varieties, seven foliar treatments, and three replicates. No treatment means or other aggregated treatment summaries were used to calculate the correlation coefficients. Spearman’s rank correlation was selected because it does not require strict normality and is suitable for evaluating monotonic relationships among traits measured on different scales. Correlation coefficients and associated *P* values were calculated using the Hmisc package [41], and the correlation matrix was visualized as a circle-based correlation plot using the corrplot package [42]. Only significant correlations at *P* ≤ 0.05 were displayed in the final figure to improve readability and focus on statistically supported trait associations.

A clustered heatmap was generated using Z-score-standardized treatment means to summarize the relative response patterns across flax varieties and foliar treatments. For each trait, the treatment means were centered and scaled so that positive Z-scores represented values above the overall mean of that trait, whereas negative Z-scores represented values below the overall mean. This standardization allowed traits measured in different units to be compared within the same visualization. Hierarchical clustering was applied to both treatment combinations and traits, and the heatmap was generated using the *pheatmap* package [43]. The Z-score-transformed means were used only for descriptive visualization and were not used in the correlation or inferential statistical analyses.

## Conclusion

Foliar application of β-carotene and carrot root extract was associated with improvements in several growth, physiological, biochemical, and yield-related traits of flax grown in sandy soil under a field-imposed short-term water-deficit episode. The magnitude and direction of the responses varied according to genotype, treatment concentration, and the measured trait. Carrot root extract at 20% produced the highest shoot length and shoot fresh weight, whereas β-carotene at 75 mg L⁻¹ was particularly effective for root elongation. Carrot root extract at 10% produced the most consistent overall response across photosynthetic pigments, IAA concentration, antioxidant-related traits, seed yield, and oil yield. β-Carotene at 50 mg L⁻¹ was also associated with marked increases in biological yield, seed yield, and oil yield. These findings suggest that carrot root extract and β-carotene may represent promising foliar treatments for supporting flax performance under the conditions evaluated in the present study; however, their effects were concentration-, genotype-, and trait-dependent. Future studies should evaluate these treatments under quantitatively defined irrigation regimes and across different soil and environmental conditions. Measurements of soil moisture, plant water status, gas exchange, chlorophyll fluorescence, oxidative-stress indicators, and antioxidant enzyme activities, together with detailed identification and quantification of the individual constituents of carrot root extract, are required to clarify the mechanisms associated with the observed responses and to confirm the optimum application concentrations.

## Supplementary Information

Supplementary Material 1.

## Data Availability

The datasets generated and/or analyzed during the current study are available from the corresponding author on reasonable request.
