# Supplementary material for: Physiological effects of foliar-applied β-carotene and carrot root extract on growth, biochemical traits, and yield of two flax varieties grown in sandy soil
Source: BMC Plant Biol. 2026 Aug 18;26:1420. doi: 10.1186/s12870-026-09711-3 (PMC13488247; doi:10.1186/s12870-026-09711-3)
Supplement: Supplementary file 1 [file 12870_2026_9711_MOESM1_ESM.docx]

| *ShootL* | | | | | |
| --- | --- | --- | --- | --- | --- |
| SOV | Df | Sum Sq | Mean Sq | F value | *p* |
| Rep | 2 | 24.490 | 12.245 | 0.465 | .634 |
| **Var** | **1** | **1,774.890** | **1,774.890** | **40.778** | **.024*** |
| Ea | 2 | 87.052 | 43.526 |  |  |
| **Foliar** | **6** | **1,811.620** | **301.937** | **11.456** | **< .001***** |
| **Var:Foliar** | **6** | **789.140** | **131.523** | **4.990** | **.002**** |
| Eb | 24 | 632.538 | 26.356 |  |  |

| *ShootFW* | | | | | |
| --- | --- | --- | --- | --- | --- |
| SOV | Df | Sum Sq | Mean Sq | F value | *p* |
| Rep | 2 | 0.322 | 0.161 | 1.211 | .315 |
| **Var** | **1** | **10.440** | **10.440** | **84.081** | **.012*** |
| Ea | 2 | 0.248 | 0.124 |  |  |
| **Foliar** | **6** | **21.830** | **3.638** | **27.379** | **< .001***** |
| **Var:Foliar** | **6** | **9.396** | **1.566** | **11.785** | **< .001***** |
| Eb | 24 | 3.189 | 0.133 |  |  |

| *RootL* | | | | | |
| --- | --- | --- | --- | --- | --- |
| SOV | Df | Sum Sq | Mean Sq | F value | *p* |
| Rep | 2 | 0.141 | 0.070 | 0.957 | .398 |
| **Var** | **1** | **38.133** | **38.133** | **224.061** | **.004**** |
| Ea | 2 | 0.340 | 0.170 |  |  |
| **Foliar** | **6** | **41.811** | **6.968** | **94.699** | **< .001***** |
| **Var:Foliar** | **6** | **1.907** | **0.318** | **4.319** | **.004**** |
| Eb | 24 | 1.766 | 0.074 |  |  |

| *RootFW* | | | | | |
| --- | --- | --- | --- | --- | --- |
| SOV | Df | Sum Sq | Mean Sq | F value | *p* |
| Rep | 2 | 0.000 | 0.000 | 0.307 | .738 |
| **Var** | **1** | **0.185** | **0.185** | **127.165** | **.008**** |
| Ea | 2 | 0.003 | 0.001 |  |  |
| **Foliar** | **6** | **0.249** | **0.042** | **145.569** | **< .001***** |
| **Var:Foliar** | **6** | **0.124** | **0.021** | **72.346** | **< .001***** |
| Eb | 24 | 0.007 | 0.000 |  |  |

| *Chl.a* | | | | | |
| --- | --- | --- | --- | --- | --- |
| SOV | Df | Sum Sq | Mean Sq | F value | *p* |
| Rep | 2 | 0.000 | 0.000 | 0.014 | .986 |
| **Var** | **1** | **0.250** | **0.250** | **54.678** | **.018*** |
| Ea | 2 | 0.009 | 0.005 |  |  |
| **Foliar** | **6** | **1.407** | **0.234** | **195.053** | **< .001***** |
| **Var:Foliar** | **6** | **0.035** | **0.006** | **4.903** | **.002**** |
| Eb | 24 | 0.029 | 0.001 |  |  |

| *Chl.b* | | | | | |
| --- | --- | --- | --- | --- | --- |
| SOV | Df | Sum Sq | Mean Sq | F value | *p* |
| Rep | 2 | 0.000 | 0.000 | 1.153 | .333 |
| **Var** | **1** | **0.017** | **0.017** | **45.776** | **.021*** |
| Ea | 2 | 0.001 | 0.000 |  |  |
| **Foliar** | **6** | **0.076** | **0.013** | **84.303** | **< .001***** |
| **Var:Foliar** | **6** | **0.021** | **0.004** | **23.499** | **< .001***** |
| Eb | 24 | 0.004 | 0.000 |  |  |

| *Carotenoides* | | | | | |
| --- | --- | --- | --- | --- | --- |
| SOV | Df | Sum Sq | Mean Sq | F value | *p* |
| Rep | 2 | 0.000 | 0.000 | 1.349 | .278 |
| **Var** | **1** | **0.003** | **0.003** | **43.806** | **.022*** |
| Ea | 2 | 0.000 | 0.000 |  |  |
| **Foliar** | **6** | **0.058** | **0.010** | **223.910** | **< .001***** |
| **Var:Foliar** | **6** | **0.017** | **0.003** | **66.189** | **< .001***** |
| Eb | 24 | 0.001 | 0.000 |  |  |

| *T.pigments* | | | | | |
| --- | --- | --- | --- | --- | --- |
| SOV | Df | Sum Sq | Mean Sq | F value | *p* |
| Rep | 2 | 0.000 | 0.000 | 0.031 | .969 |
| **Var** | **1** | **0.335** | **0.335** | **53.098** | **.018*** |
| Ea | 2 | 0.013 | 0.006 |  |  |
| **Foliar** | **6** | **2.763** | **0.460** | **210.141** | **< .001***** |
| **Var:Foliar** | **6** | **0.093** | **0.016** | **7.106** | **< .001***** |
| Eb | 24 | 0.053 | 0.002 |  |  |

| *IAA* | | | | | |
| --- | --- | --- | --- | --- | --- |
| SOV | Df | Sum Sq | Mean Sq | F value | *p* |
| Rep | 2 | 0.009 | 0.004 | 0.001 | .999 |
| **Var** | **1** | **870.324** | **870.324** | **188.579** | **.005**** |
| Ea | 2 | 9.230 | 4.615 |  |  |
| **Foliar** | **6** | **2,639.481** | **439.913** | **60.500** | **< .001***** |
| **Var:Foliar** | **6** | **127.022** | **21.170** | **2.912** | **.028*** |
| Eb | 24 | 174.510 | 7.271 |  |  |

| *Oil* | | | | | |
| --- | --- | --- | --- | --- | --- |
| SOV | Df | Sum Sq | Mean Sq | F value | *p* |
| Rep | 2 | 0.276 | 0.138 | 0.918 | .413 |
| **Var** | **1** | **57.916** | **57.916** | **234.999** | **.004**** |
| Ea | 2 | 0.493 | 0.246 |  |  |
| **Foliar** | **6** | **21.016** | **3.503** | **23.333** | **< .001***** |
| **Var:Foliar** | **6** | **2.980** | **0.497** | **3.309** | **.016*** |
| Eb | 24 | 3.603 | 0.150 |  |  |

| *Phenolic* | | | | | |
| --- | --- | --- | --- | --- | --- |
| SOV | Df | Sum Sq | Mean Sq | F value | *p* |
| Rep | 2 | 0.208 | 0.104 | 0.008 | .992 |
| **Var** | **1** | **197.383** | **197.383** | **243.750** | **.004**** |
| Ea | 2 | 1.620 | 0.810 |  |  |
| **Foliar** | **6** | **2,353.210** | **392.202** | **30.053** | **< .001***** |
| **Var:Foliar** | **6** | **251.821** | **41.970** | **3.216** | **.018*** |
| Eb | 24 | 313.210 | 13.050 |  |  |

| *Flavonoids* | | | | | |
| --- | --- | --- | --- | --- | --- |
| SOV | Df | Sum Sq | Mean Sq | F value | *p* |
| Rep | 2 | 2.971 | 1.485 | 0.374 | .692 |
| **Var** | **1** | **0.502** | **0.502** | **33.143** | **.029*** |
| Ea | 2 | 0.030 | 0.015 |  |  |
| **Foliar** | **6** | **415.722** | **69.287** | **17.428** | **< .001***** |
| **Var:Foliar** | **6** | **94.760** | **15.793** | **3.973** | **.007**** |
| Eb | 24 | 95.416 | 3.976 |  |  |

| *B-Carotene* | | | | | |
| --- | --- | --- | --- | --- | --- |
| SOV | Df | Sum Sq | Mean Sq | F value | *p* |
| Rep | 2 | 0.000 | 0.000 | 1.153 | .333 |
| **Var** | **1** | **0.001** | **0.001** | **147.092** | **.007**** |
| Ea | 2 | 0.000 | 0.000 |  |  |
| **Foliar** | **6** | **0.021** | **0.004** | **29.183** | **< .001***** |
| **Var:Foliar** | **6** | **0.006** | **0.001** | **8.942** | **< .001***** |
| Eb | 24 | 0.003 | 0.000 |  |  |

| *DPPH* | | | | | |
| --- | --- | --- | --- | --- | --- |
| SOV | Df | Sum Sq | Mean Sq | F value | *p* |
| Rep | 2 | 3.078 | 1.539 | 2.231 | .129 |
| **Var** | **1** | **1,230.234** | **1,230.234** | **91.858** | **.011*** |
| Ea | 2 | 26.786 | 13.393 |  |  |
| **Foliar** | **6** | **155.338** | **25.890** | **37.534** | **< .001***** |
| **Var:Foliar** | **6** | **20.104** | **3.351** | **4.858** | **.002**** |
| Eb | 24 | 16.554 | 0.690 |  |  |

| *PAL* | | | | | |
| --- | --- | --- | --- | --- | --- |
| SOV | Df | Sum Sq | Mean Sq | F value | *p* |
| Rep | 2 | 0.000 | 0.000 | 0.026 | .975 |
| **Var** | **1** | **1.137** | **1.137** | **68.545** | **.014*** |
| Ea | 2 | 0.033 | 0.017 |  |  |
| **Foliar** | **6** | **6.714** | **1.119** | **464.523** | **< .001***** |
| **Var:Foliar** | **6** | **0.254** | **0.042** | **17.563** | **< .001***** |
| Eb | 24 | 0.058 | 0.002 |  |  |

| *TAL* | | | | | |
| --- | --- | --- | --- | --- | --- |
| SOV | Df | Sum Sq | Mean Sq | F value | *p* |
| Rep | 2 | 0.004 | 0.002 | 0.122 | .886 |
| **Var** | **1** | **14.104** | **14.104** | **98.092** | **.010*** |
| Ea | 2 | 0.288 | 0.144 |  |  |
| **Foliar** | **6** | **8.665** | **1.444** | **92.326** | **< .001***** |
| **Var:Foliar** | **6** | **0.964** | **0.161** | **10.272** | **< .001***** |
| Eb | 24 | 0.375 | 0.016 |  |  |

| *PH* | | | | | |
| --- | --- | --- | --- | --- | --- |
| SOV | Df | Sum Sq | Mean Sq | F value | *p* |
| Rep | 2 | 30.493 | 15.247 | 1.344 | .280 |
| **Var** | **1** | **137.596** | **137.596** | **42.413** | **.023*** |
| Ea | 2 | 6.488 | 3.244 |  |  |
| **Foliar** | **6** | **2,332.706** | **388.784** | **34.270** | **< .001***** |
| **Var:Foliar** | **6** | **1,042.614** | **173.769** | **15.317** | **< .001***** |
| Eb | 24 | 272.271 | 11.345 |  |  |

| *TSL* | | | | | |
| --- | --- | --- | --- | --- | --- |
| SOV | Df | Sum Sq | Mean Sq | F value | *p* |
| Rep | 2 | 13.780 | 6.890 | 1.327 | .284 |
| **Var** | **1** | **176.013** | **176.013** | **37.185** | **.026*** |
| Ea | 2 | 9.467 | 4.734 |  |  |
| **Foliar** | **6** | **1,345.381** | **224.230** | **43.178** | **< .001***** |
| **Var:Foliar** | **6** | **357.697** | **59.616** | **11.480** | **< .001***** |
| Eb | 24 | 124.635 | 5.193 |  |  |

| *FZL* | | | | | |
| --- | --- | --- | --- | --- | --- |
| SOV | Df | Sum Sq | Mean Sq | F value | *p* |
| Rep | 2 | 1.398 | 0.699 | 0.659 | .526 |
| **Var** | **1** | **2.376** | **2.376** | **48.950** | **.020*** |
| Ea | 2 | 0.097 | 0.049 |  |  |
| **Foliar** | **6** | **538.173** | **89.696** | **84.614** | **< .001***** |
| **Var:Foliar** | **6** | **556.684** | **92.781** | **87.524** | **< .001***** |
| Eb | 24 | 25.441 | 1.060 |  |  |

| *NFBP* | | | | | |
| --- | --- | --- | --- | --- | --- |
| SOV | Df | Sum Sq | Mean Sq | F value | *p* |
| Rep | 2 | 0.459 | 0.230 | 0.171 | .844 |
| **Var** | **1** | **22.866** | **22.866** | **280.039** | **.004**** |
| Ea | 2 | 0.163 | 0.082 |  |  |
| **Foliar** | **6** | **180.615** | **30.103** | **22.348** | **< .001***** |
| **Var:Foliar** | **6** | **173.704** | **28.951** | **21.493** | **< .001***** |
| Eb | 24 | 32.328 | 1.347 |  |  |

| *NCP* | | | | | |
| --- | --- | --- | --- | --- | --- |
| SOV | Df | Sum Sq | Mean Sq | F value | *p* |
| Rep | 2 | 8.411 | 4.206 | 0.307 | .738 |
| **Var** | **1** | **914.573** | **914.573** | **77.043** | **.013*** |
| Ea | 2 | 23.742 | 11.871 |  |  |
| **Foliar** | **6** | **3,241.889** | **540.315** | **39.501** | **< .001***** |
| **Var:Foliar** | **6** | **241.564** | **40.261** | **2.943** | **.027*** |
| Eb | 24 | 328.287 | 13.679 |  |  |

| *SYP* | | | | | |
| --- | --- | --- | --- | --- | --- |
| SOV | Df | Sum Sq | Mean Sq | F value | *p* |
| Rep | 2 | 0.007 | 0.003 | 0.023 | .977 |
| **Var** | **1** | **3.276** | **3.276** | **30.431** | **.031*** |
| Ea | 2 | 0.215 | 0.108 |  |  |
| **Foliar** | **6** | **14.324** | **2.387** | **15.744** | **< .001***** |
| **Var:Foliar** | **6** | **9.058** | **1.510** | **9.957** | **< .001***** |
| Eb | 24 | 3.639 | 0.152 |  |  |

| *BYP* | | | | | |
| --- | --- | --- | --- | --- | --- |
| SOV | Df | Sum Sq | Mean Sq | F value | *p* |
| Rep | 2 | 0.214 | 0.107 | 0.682 | .515 |
| **Var** | **1** | **8.158** | **8.158** | **41.403** | **.023*** |
| Ea | 2 | 0.394 | 0.197 |  |  |
| **Foliar** | **6** | **60.078** | **10.013** | **63.715** | **< .001***** |
| **Var:Foliar** | **6** | **2.591** | **0.432** | **2.748** | **.035*** |
| Eb | 24 | 3.772 | 0.157 |  |  |

| *SI* | | | | | |
| --- | --- | --- | --- | --- | --- |
| SOV | Df | Sum Sq | Mean Sq | F value | *p* |
| Rep | 2 | 0.241 | 0.120 | 1.004 | .381 |
| **Var** | **1** | **2.140** | **2.140** | **59.078** | **.017*** |
| Ea | 2 | 0.072 | 0.036 |  |  |
| **Foliar** | **6** | **13.913** | **2.319** | **19.325** | **< .001***** |
| **Var:Foliar** | **6** | **2.990** | **0.498** | **4.153** | **.005**** |
| Eb | 24 | 2.880 | 0.120 |  |  |

| *StYH* | | | | | |
| --- | --- | --- | --- | --- | --- |
| SOV | Df | Sum Sq | Mean Sq | F value | *p* |
| Rep | 2 | 0.005 | 0.003 | 0.012 | .988 |
| **Var** | **1** | **1.604** | **1.604** | **56.003** | **.017*** |
| Ea | 2 | 0.057 | 0.029 |  |  |
| **Foliar** | **6** | **109.921** | **18.320** | **82.977** | **< .001***** |
| **Var:Foliar** | **6** | **14.477** | **2.413** | **10.928** | **< .001***** |
| Eb | 24 | 5.299 | 0.221 |  |  |

| *SYH* | | | | | |
| --- | --- | --- | --- | --- | --- |
| SOV | Df | Sum Sq | Mean Sq | F value | *p* |
| Rep | 2 | 0.000 | 0.000 | 0.017 | .983 |
| **Var** | **1** | **0.533** | **0.533** | **48.977** | **.020*** |
| Ea | 2 | 0.022 | 0.011 |  |  |
| **Foliar** | **6** | **2.681** | **0.447** | **69.520** | **< .001***** |
| **Var:Foliar** | **6** | **0.145** | **0.024** | **3.747** | **.009**** |
| Eb | 24 | 0.154 | 0.006 |  |  |

| *BYH* | | | | | |
| --- | --- | --- | --- | --- | --- |
| SOV | Df | Sum Sq | Mean Sq | F value | *p* |
| Rep | 2 | 0.231 | 0.115 | 0.472 | .629 |
| **Var** | **1** | **0.321** | **0.321** | **101.744** | **.010**** |
| Ea | 2 | 0.006 | 0.003 |  |  |
| **Foliar** | **6** | **136.225** | **22.704** | **92.911** | **< .001***** |
| **Var:Foliar** | **6** | **15.140** | **2.523** | **10.326** | **< .001***** |
| Eb | 24 | 5.865 | 0.244 |  |  |

| *OYH* | | | | | |
| --- | --- | --- | --- | --- | --- |
| SOV | Df | Sum Sq | Mean Sq | F value | *p* |
| Rep | 2 | 1,441.739 | 720.869 | 0.894 | .422 |
| **Var** | **1** | **17,990.619** | **17,990.619** | **38.220** | **.025*** |
| Ea | 2 | 941.414 | 470.707 |  |  |
| **Foliar** | **6** | **330,714.676** | **55,119.113** | **68.356** | **< .001***** |
| **Var:Foliar** | **6** | **14,434.471** | **2,405.745** | **2.983** | **.025*** |
| Eb | 24 | 19,352.539 | 806.356 |  |  |
